# Aligning the many definitions of treatment resistance in anxiety disorders: A systematic review

**DOI:** 10.1002/da.22895

**Published:** 2019-06-23

**Authors:** Wicher A. Bokma, Guido A. A. M. Wetzer, Jurriaan B. Gehrels, Brenda W. J. H. Penninx, Neeltje M. Batelaan, Anton L. J. M. van Balkom

**Affiliations:** ^1^ Department of Psychiatry, Amsterdam UMC Vrije Universiteit, Psychiatry, Amsterdam Public Health Research Institute Amsterdam The Netherlands; ^2^ GGZ inGeest Specialized Mental Health Care Amsterdam The Netherlands

**Keywords:** anxiety disorders, assessment/diagnosis, CBT/ cognitive behavioral therapy, pharmacotherapy, treatment resistance

## Abstract

Anxiety Disorders often show a chronic course, even when treated with one of the various effective treatments available. Lack of treatment effect could be due to Treatment Resistance (TR). Consensus on a definition for TR Anxiety Disorders (TR‐AD) is highly needed as currently many different operationalizations are in use. Therefore, generalizability in current TR‐AD research is suboptimal, hampering improvement of clinical care. The objective of this review is to evaluate the currently used definitions of TR‐AD by performing a systematic review of available literature. Out of a total of *n* = 13 042, 62 studies that operationalized TR‐AD were included. The current review confirms a lack of consensus on TR‐AD criteria. In 62.9% of the definitions, TR was deemed present after the first treatment failure. Most studies (93.0%) required pharmacological treatment failures, whereas few (29.0%) required psychological treatment failures. However, criteria for what constitutes “treatment failure” were not provided in the majority of studies (58.1%). Definitions for minimal treatment duration ranged from at least 4 weeks to at least 6 months. Almost half of the TR‐AD definitions (46.8%) required elevated anxiety severity levels in TR‐AD. After synthesis of the results, the consensus definition considers TR‐AD present after both at least one first‐line pharmacological and one psychological treatment failure, provided for an adequate duration (at least 8 weeks) with anxiety severity remaining above a specified threshold. This definition could contribute to improving course prediction and identifying more targeted treatment options for the highly burdened subgroup of TR‐AD patients.

## 1 INTRODUCTION

Up till now, a widely used definition for treatment resistance in anxiety disorders does not exist (Barton, Karner, Salih, Baldwin, & Edwards, 2014; Chen & Tsai, 2016; Roy‐Byrne, 2015; Starcevic, 2008; Stein, 2004). This is surprising because it is well known that a substantial proportion of adults with Anxiety Disorders experience suboptimal treatment results after evidence‐based treatments (Bruce et al., 2005; Huh, Goebert, Takeshita, Lu, & Kang, 2011; Ramsawh, Raffa, Edelen, Rende, & Keller, 2009). Selective Serotonin Reuptake Inhibitors (SSRIs) and Serotonin Norepinephrine Reuptake Inhibitors (SNRIs) are widely regarded the first‐line pharmacological treatments for Anxiety Disorders (Bandelow et al., 2008; Bandelow et al., 2012; Farach et al., 2012). Cognitive Behavioural Therapy (CBT) is the first‐line psychological treatment option for Anxiety Disorders (Taylor, Abramowitz, & McKay, 2012). First‐line treatments show a moderate effect size in meta‐analytic comparisons with placebo (Carpenter et al., 2018; Jakubovski, Johnson, Nasir, Müller‐Vahl, & Bloch, 2018). After first‐line treatment up till 30–60% of patients have substantial and impairing remaining symptoms (Bandelow et al., 2008; Bruce et al., 2005; Tyrer, Seivewright, & Johnson, 2004).

A wide variety of terms are in use for the phenomenon of suboptimal treatment results in anxiety disorders: “refractory anxiety”, “treatment resistance”, “medication resistance”, “treatment refractory cases”, “remaining symptomatic” and “persistent symptoms” (Baldwin, Stein, & Hermann, 2016; Bystritsky, Stein, & Hermann, 2016; Craske, Bystritsky, Stein, & Hermann, 2016; Craske, Stein, & Hermann, 2016; Ipser et al., 2006; Roy‐Byrne, Stein, & Hermann, 2016). In other psychiatric disorders the term “treatment resistance” (TR) is preferred to describe a subgroup of patients who have a prior history of unfavorable treatment effects (Conway, George, & Sackeim, 2017; Fekadu et al., 2009; Fogelson & Leuchter, 2017; Howes et al., 2016), which also implies having less favorable future treatment effects (Conway et al., 2017; Fekadu et al., 2009; Fogelson & Leuchter, 2017; Howes et al., 2016). The varying terminology reflects the absence of consensus regarding the *criteria* for TR (Barton et al., 2014; Cirillo, Freire, & Freire, 2016; Perna & Caldirola, 2017; Stein & Seedat, 2004; Yoshinaga et al., 2016). This lack of consensus on criteria for TR‐AD was first recognized in 2004; however, 14 years later still no consensus exists (Barton et al., 2014; Chen & Tsai, 2016; Roy‐Byrne, 2015; Starcevic, 2008; Stein & Seedat, 2004; Van Ameringen & Mancini, 2001).

Most authors define Treatment Resistant Anxiety Disorders (TR‐AD) as the persistence of anxiety symptoms, or as the absence of response, recovery or remission of the disorder after some form of active treatment (Barton et al., 2014; Davies, Dubovsky, Gabbert, & Champman, 2000; Holt & Lydiard, 2007; Pollack, Smoller, Otto, & Rosenbaum, 1996; Stein, 2004; Zoun et al., 2016). These active treatments should represent evidence‐based treatment regimes, provided at an adequate dosage and for an adequate duration (Fava, Rafanelli, & Tomba, 2012; Roy‐Byrne, 2015). However, the absence of anxiety symptoms does not always indicate full disorder remission (Bystritsky, 2006; Chen & Tsai, 2016). A substantial amount of residual disease burden may be present in persisting behavioural changes such as avoidance, or in altered cognitive functioning, for instance in excessive rumination. Additional emphasis on functional recovery is therefore advocated by a number of authors when assessing TR‐AD (Bystritsky, 2006; Chen & Tsai, 2016). No systematic review into the definition for TR‐AD is yet performed.

The aim of this study is to summarize and discuss the different criteria used for TR‐AD. To do this, we will perform a systematic literature review. Second, by summarizing and comparing the different criteria used for TR in anxiety disorders, we aim to propose a consensus definition for TR‐AD.

## 2 METHODS

The methods for this systematic review were specified in advance in a study protocol which was documented in the PROSPERO database (reference number CRD42017055864). The current paper was drafted in accordance with the PRISMA guidelines for reporting on systematic reviews (Liberati et al., 2009).

### 2.1 Literature search

A systematic search across MEDLINE, PubMed (non‐MEDLINE), EMBASE, PsycINFO, and Web of Science for available literature until April 2018 was performed. To derive all articles that might include a definition for TR in anxiety disorders we searched for Anxiety Disorders (according to DSM‐5, American Psychiatric Association, 2013) in combination with various free‐text synonyms for “treatment resistance” (see Panel 1) for the full search query).

**Panel 1:** Overview of search terms used in this systematic review (formatted for MEDLINE)

(("Anxiety Disorders"[Mesh:NoExp] OR "Agoraphobia"[Mesh] OR "Anxiety, Separation"[Mesh] OR "Neurocirculatory Asthenia"[Mesh] OR "Neurotic Disorders"[Mesh] OR "Panic Disorder"[Mesh] OR "Phobic Disorders"[Mesh] OR anxiety disorder* [tiab] OR generalized anxiety disorder* [tiab] OR generalised anxiety disorder* [tiab] OR anxiety state* [tiab] OR agoraphobi* [tiab] OR panic* [tiab] OR phobi* [tiab] OR selective mutis* [tiab]))
AND
(“Retreatment” [Mesh] OR "Drug Resistance" [Mesh:NoExp] OR “Drug tolerance” [Mesh] OR treatment resistan* [tiab] OR refractor* [tiab] OR poor respon* [tiab] OR partial respon* [tiab] OR non‐respon* [tiab] OR nonrespon* [tiab] OR loss of respons* [tiab] OR medication resistan* [tiab] OR drug resistan* [tiab] OR tachyphyl* [tiab] OR resilien* [tiab] OR persistan* [tiab] OR immune [tiab] OR insusceptib* [tiab] OR irresponsive* [tiab] OR unreceptive* [tiab] OR resistive [tiab] OR unsuccessful treatment* [tiab] OR treatment failur* [tiab] OR failed treatment* [tiab] OR "Patient Dropouts"[Mesh] OR patient dropout* [tiab] OR treatment dropout* [tiab] OR "Patient Compliance"[Mesh] OR non‐complian* [tiab] OR noncomplian* [tiab] OR non‐adheren* [tiab] OR nonadheren* [tiab] OR remaining symptom* [tiab] OR pseudo‐resistan* [tiab] OR dropping out [tiab] OR augmentation [tiab] OR inadequate respon* [tiab] OR intractab* [tiab] OR partially respon* [tiab] OR resistant patient* [tiab] OR remain symptom* [tiab] OR remaining symptom* [tiab] OR non‐remitting [tiab] OR nonremitting [tiab] OR partial improvement* [tiab] OR incomplete respon* [tiab] OR residual symptom* [tiab] OR anxiolytic toleran* [tiab])

All publication types in English were included with the exception of conference summaries, editorials, columns, book reviews and manifestos as these were unlikely to include a full description of a TR‐AD definition. Studies were selected when they included adults or elderly persons with anxiety disorders (Panic Disorder (with or without Agoraphobia, PD(A)), Social Anxiety Disorder (SAD), Generalized Anxiety Disorder (GAD), Specific Phobia (SP), Selective Mutism, and Separation Anxiety). No restrictions in presence of comorbidity were used. Exclusion criteria included studies with an average study population below 21 years, and studies reporting primarily on posttraumatic stress disorder (PTSD) or Obsessive‐Compulsive Disorder (OCD), because these are no longer classified as Anxiety Disorders.

### 2.2 Eligibility assessment

**Figure 1:**
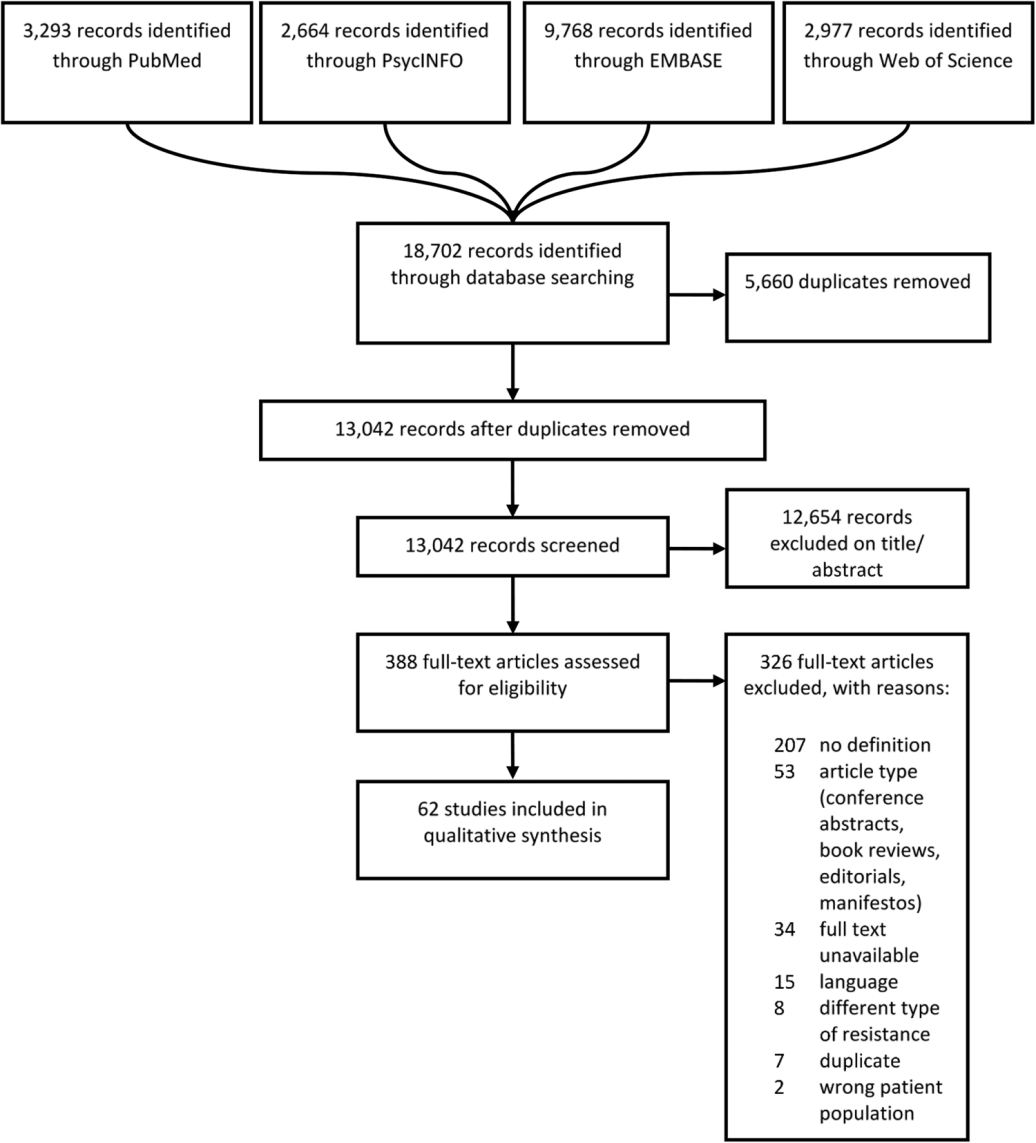
PRISMA flow chart for study inclusion

Eligibility assessment on title and abstract was performed independently by two reviewers (WB, GW, JG) by using the Cochrane‐supported review program Covidence (www.covidence.org). Disagreements were resolved by consensus after discussion. A flow chart for inclusion of eligible studies according to PRISMA guidelines is provided in Figure 1. Full‐text screening was performed independently by two reviewers (WB and JG). During the full‐text screening phase, articles were excluded if a full‐text version could not be retrieved or if any of the exclusion criteria were present. Studies were included if their definition for TR‐AD could be implicitly deduced from inclusion criteria used in a study. Reviews, meta analyses and book chapters were included if they provided their own definition for TR‐AD but were excluded if they repeated other studies’ definitions without providing rationale for choosing this definition over others. As the vast majority of studies used TR and “refractory” interchangeably we chose to regard them as synonyms and will refer to these phenomena as TR‐AD.

### 2.3 Data extraction

From trials we extracted data on study characteristics: number of subjects, population of interest, intervention, comparator condition, follow‐up period, primary outcomes and results; from reviews we extracted data on study design and population of interest. With regard to the definitions for TR‐AD, we extracted data on nine predefined putative criteria for the definition, based on criteria used in the Maudsley Staging Method for treatment resistant depressive disorders (Fekadu et al., 2009). In addition, we extracted one TR‐AD criterion (treatment response), that was not predefined in our study protocol. The ten criteria were: minimal number of failed treatments, failed psychotherapy trials, failed pharmacological trials, failed other biological treatments, minimal length of treatment, treatment response criterion (i.e., which posttreatment change constitutes response/failure), minimal duration of anxiety disorder, severity of symptoms, presence of functional impairment, and presence of comorbidity. We evaluated which of these ten criteria were present in TR‐AD definitions across included studies (yes/no). Specific values for each criterion were extracted as well.

### 2.4 Quality of definitions

We assessed the definition quality in each included study. As there are no formal risk of bias tools available for the purpose of our study, and as we are not interested in potential sources of study outcome bias we assessed definition quality in two ways; first, by counting the total number of TR‐AD criteria included in each study's definition, second, by determining the degrees of precision with which the definition for TR‐AD is presented in each paper. The total number of TR‐AD criteria was a count variable counting presence of all ten dichotomized TR‐AD criteria. Degrees of precision was categorized into “high”, “medium” and “low”. Precision was considered “high” if a study provided an explicit definition for TR‐AD, for example in this study by De Salas‐Cansado et al. (2013):

*Refractory was defined as subjects with persistent symptoms/suboptimal response, a Hamilton‐anxiety (HAM‐A) scale score ≥ 16 and a Clinic Global Impression (CGI) score ≥ 3 at baseline, after a standard dose regimen of any anti‐anxiety drug, alone or in combination, for at least 6 months, given before the baseline study visit.* (p987).

The degree of precision was deemed “medium” if the criteria were only implicitly attributable to the concept of TR‐AD, or if multiple terms were used interchangeably, for instance in a study by Lohoff, Etemad, Mandos, Gallop, and Rickels (2010) in patients with “refractory GAD”:

*Subjects also had to have treatment failure of at least 1 adequate trial of an SSRI, an SNRI, a BZ, or a combination of these agents. Patients who were on an SSRI, an SNRI, a BZ, or a combination of these agents before enrollment had to be on a stable dose for 4 weeks. Inclusion further required a total score of 16 or higher on the Hamilton Anxiety Scale (HAM‐A) and a score of 4 or greater on the Clinical Global Impression Severity of Illness Scale* (CGI‐S) (p186).

Finally, if the study only provided a description of the concept of TR‐AD, without operationalizing it in specific criteria, the degree of precision was deemed “low”, for instance:

“*failure of an adequate clinical trial of medication*” (Stein, 2004).

### 2.5 Data synthesis

To synthesize the results of the systematic review into a new operationalization for TR‐AD, frequencies for presence of each individual TR‐AD criterion were assessed. The most frequently used values for each individual criterion were considered the most appropriate operationalization for that criterion and were chosen for the consensus definition. However, if an unspecified category for a certain criterion (e.g., “unspecified type of pharmacological treatment”) was the most frequently used value, we did not consider this category for the new definition if a more specified value was available. In addition, criteria that were included only in a small minority (<10%) of the studies were not used for the new definition, as they were then judged to be lacking a convincing empirical basis.

### 2.6 Statistical analyses

To test associations between total number of criteria provided in definitions, degrees of precision and publication year, we performed Kruskall‐Wallis tests for differences in mean rank. We hypothesized that higher definition quality studies (i.e., more total criteria or a higher degree of precision) would be the most recent studies. Fisher's Exact tests were performed to investigate whether the two definition quality variables were associated with different frequencies for values of each TR‐AD criterion. For instance: did high definition quality studies more often require a higher number of failed treatments or more often mention a SSRI/SNRI failure as requisite for TR‐AD compared with lower definition quality studies?

## 3 RESULTS

### 3.1 Study selection

The electronic database search yielded 18,702 results. After deduplication 13,042 entries remained. During title and abstract screening, 12,654 studies were excluded. We assessed 388 full‐text studies, of which 207 did not contain a definition, 53 were a wrong article type (conference abstracts and editorials), 34 could not be retrieved, 15 did not meet language requirements, 8 reported on a different type of “resistance” (e.g., “resistance” in the psychodynamic paradigm), 7 were previously unrecognized duplicates and 2 reported on a different patient population. This resulted in the final inclusion of 62 studies (for a flow chart see Figure 1).

### 3.2 Characteristics of included studies

Included studies were published between 1986 and 2018. They consisted of eight narrative reviews (Bakker, Van Balkom, & Stein, 2005; Bandelow et al., 2008; Bystritsky, 2006; Chen & Tsai, 2016; Holt & Lydiard, 2007; Lorenz, Jackson, & Saitz, 2010; Pollack, 2009; Starcevic, 2008), five systematic reviews (Barton et al., 2014; Cosci & Fava, 2013; Ipser et al., 2006; Patterson & Van Ameringen, 2016; Samuel, Zimovetz, Gabriel, & Beard, 2011), of which three also performed meta‐analyses (Barton et al., 2014; Ipser et al., 2006; Patterson & Van Ameringen, 2016), seven treatment guidelines/algorithms (Bandelow, 2008; Bandelow, Zohar, Hollander, Kasper, & Moller, 2002; National Institute for Health & Clinical Excellence, 2011; Stein et al., 2001; Stein et al., 2010; Stein, 2003; Stein, 2004), three book chapters (Baldwin, Polkinghorn, Lerer, & Stahl, 2005; Deligiannidis & Rothschild, 2010; Van Ameringen, Mancini, Patterson, & Stein, 2009), 21 open‐label trials (Gabriel & Violato, 2011; Gabriel, 2010; George et al., 2008; Glue et al., 2017, 2018; Heldt et al., 2003; Heldt et al., 2006; Hoge et al., 2008; Hollifield, Thompson, Ruiz, & Uhlenhuth, 2005; Katzman et al., 2008; Kinrys, Vasconcelos E Sa, & Nery, 2007; Menza, Dobkin, & Marin, 2007; Ociskova, Prasko, Latalova, Kamaradova, & Grambal, 2016; Pallanti & Quercioli, 2006; Pollack, Otto, Kaspi, Hammerness, & Rosenbaum, 1994; Simon et al., 2006; Snyderman et al., 2005; Solbakken & Abbass, 2015, 2016; Worthington III, Kinrys, Wygant, & Pollack, 2005; Yoshinaga et al., 2016), eight RCTs (Brawman‐Mintzer, Knapp, & Nietert, 2005; Castle, Gray, Neehoff, & Glue, 2017; Gloster et al., 2015; Hirschmann et al., 2000; Lohoff et al., 2010; Pollack et al., 2006; Rickels et al., 2012; Simon et al., 2009), four retrospective cohorts (Bakish et al., 1995; Cowley, Ha, & Roy‐Byrne, 1997; Durham, Higgins, Chambers, Swan, & Dow, 2012; Kinrys et al., 2007), one prospective cohort (Milrod et al., 2016), three case series (Aarre, 2003; Otto, Pollack, Penava, & Zucker, 1999; Tesar & Rosenbaum, 1986), one cost‐effectiveness analysis (De Salas‐Cansado et al., 2013), and one trial protocol (Zoun et al., 2016). Thirty‐three studies pertained to PD, 34 to GAD, 21 to SAD, two to SP, and five to Anxiety Disorders in general. For a summary of study characteristics see Table 1. For full details, see eTable 1 (trials, cohort studies and meta‐analyses) and eTable 2 (reviews, treatment guidelines and book chapters).

**Table 1:** Study characteristics for included studies

Study characteristics	n	%
Publication type		
Book chapter	3	4.8
Case series	3	4.8
Cost‐effectiveness analysis	1	1.6
Narrative review	8	12.9
Open‐label trial	21	33.9
Prospective cohort study	1	1.6
Randomized controlled trial	8	12.9
Retrospective cohort study	4	6.5
Systematic review	2	3.2
Systematic review + meta‐analysis	3	4.8
Trial protocol	1	1.6
Treatment guidelines/algorithms	7	11.3
Population of interest^a^		
Anxiety disorders (in general)	5	8.1
Generalized anxiety disorder	34	54.8
Panic disorder	33	53.2
Social anxiety disorder	21	33.9
Specific phobia	5	8.1
Type of intervention used (if any)		
Adjunctive psychotherapy	8	12.9
Any therapy	1	1.6
Any adjunctive therapy	1	1.6
Combination treatment: pharmacological and psychological	4	6.5
Either pharmacologic monotherapy or pharmacologic augmentation therapy	1	1.6
Pharmacologic augmentation or combination treatment	17	27.4
Pharmacologic monotherapy	7	11.3
Nerve vagus stimulation	1	1.6
Self‐management	1	1.6
Degree of precision of included definitions		
High	13	21.0
Medium	44	71.0
Low	5	8.1

^a^ Some studies described more than one population of interest.

### 3.3 Definition quality

The total number of criteria per study ranged from one to six (mean = 3.58; *SD* = 1.31). With respect to the assessment of the degree of precision for TR‐AD definitions it appeared that 13 studies (21.0%) provided a high degree of precision, 44 (71.0%) a medium degree, and 5 (8.1%) a low degree of precision.

There was a significant association between total number of criteria and year of publication (*χ* ^2^(*df* = 5) = 13.01; *p* = 0.02): the studies with the highest number of criteria were, on average, the most recent. For degrees of precision no association with publication date existed (*χ* ^2^(*df* = 2) = 2.13; *p* = 0.34). Neither studies with a higher total number of criteria, nor studies with a higher degree of precision provided a different perspective on the ten TR‐AD criteria. Since definition quality did not change operationalizations for TR‐AD, all studies were used in the synthesis of results.

### 3.4 Main results

By applying a systematic review approach it became apparent that a large majority of studies on the topic of TR‐AD (*n* = 207) do not provide a definition for the phenomenon of TR‐AD. Furthermore, the included studies (*n* = 62) yielded many different definitions for the concept of TR‐AD (see eTable 3 for all definitions). Trials often used the presence of one failed pharmacological treatment as an adequate definition for TR‐AD. Other studies provided additional criteria. When the frequencies for each of the ten extracted TR‐AD criteria were compared across included studies, some distinctive patterns arose (see Table 1).

**Table 2:** Criteria included in definitions for treatment resistance anxiety disorders

Treatment resistance definition criteria	n	%
Minimal number of failed treatments		
Not part of definition	9	14.5
Included in definition	53	85.5
Unspecified or varying number	7	11.3
1 failed treatment	39	62.9
2 failed treatments	3	4.8
3 or more failed treatments	4	6.5
Failed psychotherapy trials		
Not part of definition	44	71.0
Included in definition	18	29.0
Any	9	14.5
At least one failed CBT trial	7	11.3
Varying number (stepped‐care or staging approach)	2	3.2
Failed pharmacological trials		
Not part of definition	4	6.5
Included in definition	58	93.5
Unspecified number or type of failed pharmacological treatment	27	43.5
At least one failed SSRI/SNRI trial	15	24.2
At least one failed other pharmacotherapeutic trial	14	22.6
Varying number or types (stepped‐care or staging approach)	2	3.2
Other biological treatments		
Not part of definition	62	100
Included in definition	0	0
Minimal length of treatment		
Not part of definition	28	45.2
Included in definition	34	54.8
>4 weeks	8	12.9
>6 weeks	3	4.8
>8 weeks^a^	15	24.2
>11 weeks or 20 sessions of CBT	1	1.6
>12 weeks	3	4.8
>4 months	2	3.2
>6 months	2	3.2
Treatment response criterion		
Not part of definition	36	58.1
Included in definition	26	41.9
Cut‐off values for effective/ failed treatment provided^b^	26	41.9
Minimal duration of anxiety disorder		
Not part of definition	60	96.8
Included in definition	2	3.2
>1 year	1	1.6
>2 years	1	1.6
Severity of symptoms		
Not part of definition	33	53.2
Included in definition	29	46.8
Aspecific criterion (e.g., “severe”)	1	1.6
Specific criterion (cut‐off values) provided^c^	28	45.2
Functional impairment		
Not part of definition	57	91.9
Included in definition	5	8.1
Aspecific criterion (e.g., “marked impairments”)	4	6.5
Specific criteria (cut‐off values) provided^d^	1	1.6
Presence of comorbidity		
Not part of definition	61	98.4
Included in definition	1	1.6
Comorbidity as exclusion criterion for TR‐AD	1	1.6

*Note*: CBT: cognitive behavioral therapy; CGI‐I:Clinical Global Impression Improvement Scale; CGI‐S: Clinical Global Impression Severity Scale; HAM‐A: Hamilton Anxiety Rating Scale; LSAS: Leibowitz Social Anxiety Scale; PD(A): panic disorder (with or without agoraphobia); PDSS: Panic Disorder Severity Scale; SAD:social anxiety disorder; SDS: Sheehan Disability Scale; SSRI: selective serotonin reuptake inhibitor; SNRI: selective norepinephrine reuptake inhibitor.

^a^ including studies with minimal treatment duration of “2 months.”

^b^ the most often used criteria were: ∆HAM‐A < 50% or CGI‐I < 2.

^c^ the most often used criteria for severe symptomatology were HAM‐A < 16 or CGI‐S ≥ 4 (for all Anxiety Disorders), PDSS > 3 or any PDSS item > 1 (for PD(A)), LSAS > 60 (for SAD).

^d^ one study used SDS > 1 on each item as criterion for functional impairments.

The minimal number of required failed treatments, regardless of treatment type, was reasonably consistent across studies: 39 studies (62.9%) required one treatment failure for TR‐AD, with other studies varying between two (*n* = 3) and five (*n* = 1) failed previous treatments. Failed psychotherapy trials were only included in 18 studies (29.0%). These studies all regarded CBT an appropriate treatment, with seven studies (11.3%) restricting TR‐AD to CBT failure alone, whilst others (*n* = 9; 14.5%) also regarded other psychological treatments appropriate.

Contrastingly, a large majority (*n* = 58; 93.5%) required at least one failed pharmacotherapy trial for their definition for TR‐AD. Of these, some studies (*n* = 15; 24.2%) considered at least one failed SSRI/SNRI trial sufficient to be classified as TR‐AD. A substantial number of studies did not specify type of pharmacotherapeutic treatment failure required for TR‐AD (*n* = 27; 43.5%), for instance by referring to “first‐line” or “standard” antianxiety treatments. A few used a varying number of treatment types in a stepped‐care or staging algorithm (*n* = 2; 3.2%) or considered other pharmacotherapeutic treatment failures adequate (*n* = 14, 22.6%). See eTable 3 for detailed descriptions of types of pharmacotherapy. Whether failed trials were caused by a lack of effect, or a lack of tolerability was usually not reported. Other biological treatments were not included in TR‐AD definitions.

Most studies (*n* = 34; 54.8%) used a minimal treatment length criterion ranging from 4 weeks to 6 months, while the most often used adequate minimal treatment duration was 8 weeks (*n* = 15; 24.2%).

A substantial number of studies (*n* = 26; 41.9%) gave a response criterion. The most commonly used cut‐off values were a <50% posttreatment improvement on the Hamilton Anxiety Rating Scale (HAM‐A) and a posttreatment Clinical Global Impression Improvement scale (CGI‐I) score greater than two (i.e., “minimal improvement”, at best). Severity of anxiety symptoms was often included in definitions (*n* = 29; 46.8%), with cut‐off scores commonly provided: a HAM‐A score of above 15 (for any Anxiety Disorder), a Clinical Global Impression Severity Scale (CGI‐S) score of four or higher (for any Anxiety Disorder), a total score above 3, or any item above 1 on the Panic Disorder Severity Scale (PDSS) for PD and a score at or above 60 on the Leibowitz Social Anxiety Scale (LSAS) for SAD. For GAD, no disorder‐specific measurement instrument was reported in TR‐AD definitions. Finally, minimal disease duration (*n* = 2; 3.2%), presence of functional impairments (*n* = 5; 8.1%) and presence of comorbidity (*n* = 1; 1.6%) were sparsely included in definitions for TR‐AD. See Table 2 for a summary per TR‐AD criterion, and eTable 4 for a full overview of included TR‐AD criteria per study.

### 3.5 Synthesis of results

To propose a consensus definition for TR‐AD that reflects the current literature, we included the most prevalent values for all criteria that were provided consistently across studies into the new TR‐AD definition. Failed SSRI/SNRI trials were most often considered as criterion for TR‐AD. Studies typically referred to SSRI/SNRI trials as “first‐line” treatment. Therefore, failure of at least one first‐line treatment (SSRI/SNRI) was included in the new definition. Although psychotherapeutic treatment failure was less often incorporated in TR‐AD definitions, CBT was usually referred to as “first‐line” psychological intervention. Therefore, first‐line psychological interventions (CBT) failure was included in the new definition. Although the most often provided criterion for minimal number of treatments was one, by including both pharmacological and psychological treatment failures into the definition, the minimal number of failed treatments in the new definition rose to at least two. A minimal adequate treatment duration of 8 weeks was included in the consensus definition. In studies that permitted psychotherapy failures as criterion for TR‐AD (*n* = 21), only five provided a minimal treatment duration criterion, ranging from 4 weeks to 20 sessions CBT (see eTable 4). Therefore, a minimal duration of 8 weeks was maintained for psychotherapy trials.

Absence of treatment response was included in the consensus definition, using the two most commonly provided cut‐off values from studies included in this review. Other biological treatments, minimal duration of Anxiety Disorder, presence of functional impairments and comorbidity were only sporadically included in TR‐AD definitions and therefore were not considered for the consensus TR‐AD definition. See Panel 2 for the full description of this consensus TR‐AD definition with most commonly used cut‐off values for each criterion.

**Panel 2:** Proposed operationalization for Treatment Resistant Anxiety Disorders (TR‐AD)

TR‐AD checklist				
Failed pharmacotherapeutic treatment				
□	At least one first‐line treatment (SSRI, SNRI)^1^			
□	pre‐to posttreatment difference in HAM‐A <50% or posttreatment CGI‐I >2			
□	treatment period of at least 8 weeks			
	
Failed psychotherapeutic treatment				
□	At least one first‐line psychotherapeutic treatment (CBT)^2^			
□	pre‐to posttreatment difference in HAM‐A <50% or posttreatment CGI‐I >2			
□	provided according to local protocols and for an adequate duration (at least>8 weeks)			
	
Current severity of anxiety symptoms				
□	GAD		HAM‐A >15	or CGI‐S > 3
□	PD		HAM‐A >15 or PDSS >3, or any item >1	or CGI‐S > 3
□	SAD		HAM‐A >15 or LSAS ≥ 60	or CGI‐S > 3

*TR‐AD is present if all six treatment boxes can be checked in addition to at least one symptom severity box*

^1^SSRIs and SNRIs are considered first‐line pharmacotherapeutic treatment options as per 2018 (Bystritsky, Stein, & Hermann, 2016; National Institute for Health and Clinical Excellence, 2011; Roy‐Byrne, Stein, & Hermann, 2016)

^2^CBT interventions are considered first‐line psychotherapeutic treatment options as per 2018 (Craske, Stein, & Hermann, 2016; National Institute for Health and Clinical Excellence, 2011)

Abbreviations: SAD=  Social Anxiety Disorder, PD=  Panic Disorder, GAD=  Generalized Anxiety Disorder, HAM‐A=  Hamilton Anxiety Rating Scale, PDSS=  Panic Disorder Severity Scale, LSAS=  Leibowitz Social Anxiety Scale, CGI‐S=  Clinical Global Impression Severity Scale, CGI‐I=  Clinical Global Impression Improvement Scale, SSRI=  Selective Serotonin Reuptake Inhibitor, SNRI=  Selective Serotonin and Norepinephrine Reuptake Inhibitor, CBT=  Cognitive Behavioral Therapy.

## 4 DISCUSSION

This paper aimed to systematically review different definitions and criteria for treatment resistant Anxiety Disorders (TR‐AD) and showed that the majority of studies do not provide a definition for TR‐AD and that consistency and consensus across TR‐AD definitions in included studies is lacking. The most frequently used definition for TR‐AD simply consists of one failed first‐line pharmacotherapy treatment. Both the lack of consensus in current TR‐AD definitions and the unclear description of TR‐AD in the most used definition make the current attempt of aligning definitions a necessity.

Out of ten putative criteria, we identified six criteria that are regularly integrated into the various different definitions for TR‐AD: minimal number of treatment failures, presence (and type) of psychological treatment failure, presence (and type) of pharmacological treatment failure, minimal treatment duration (>8 weeks), specification of a response criterion (i.e., what constitutes a “failed treatment”), and minimal symptom severity. These criteria were integrated into a consensus definition. Four putative criteria were dismissed: “minimal duration of disorder”, “other biological treatment failures”, “presence of comorbidity”, and “presence of functional impairment” due to the low frequency with which these were mentioned. In selecting the specific cut‐off values for included criteria, we opted to use the most commonly mentioned cut‐off values (the mode). Based on the most recent treatment guidelines, for the purpose of this definition we considered SSRIs and SNRIs as current first‐line pharmacotherapy options, and CBT current first‐line psychotherapy option (Bystritsky et al., 2016; Craske, Stein et al., 2016; National Institute for Health & Clinical Excellence, 2011; Roy‐Byrne et al., 2016). Furthermore, the consensus definition for TR‐AD requires both a failed pharmacotherapeutic and psychotherapeutic trial, as these were both regularly used as criterion for TR‐AD.

This is the first study to systematically assess different criteria for TR‐AD. A systematic approach was complicated by the absence of a risk of bias assessment tool for the purpose of the current study. Tools such as the Cochrane risk of bias tool for randomized studies (Higgins & Green, 2011) or the RoBANS for nonrandomized studies (Kim et al., 2013) determine the level of confidence with which the results of a certain study can be interpreted. However, the data we extracted from studies referred to the definition for treatment resistance they used, not the outcome of the study. Therefore, we chose to assess definition quality by determining the total number of criteria provided and the degree of precision with which the definition was provided. After analyzing these data, it seemed that the quality of included definitions did not impact operationalization of TR‐AD.

A limitation in this study was that although integration of the definitions was done systematically the final consensus definition could still reflect some subjective choices by the authors of the current study. Furthermore, it was apparent that some criteria might have been underreported in the included studies, for instance on minimal treatment duration in CBT. Another limitation in our methodology was the lack of studies that incorporated evaluation of pseudo‐resistance into their TR‐AD definitions. Pseudo‐resistance refers to any nonresponse in treatments that are not used to their full potential. Before treatment resistance can be deemed present, pseudo‐resistance should always be ruled out. In pharmacotherapy trials this could be due to a wrong indication, an inadequate dosage or inadequate duration (Fava et al., 2012; Roy‐Byrne, 2015). In psychotherapy trials this could be due to clinicians not following the treatment protocol or patients not being compliant with homework assignments (Roy‐Byrne, 2015; Taylor et al., 2012). In addition to this, in clinical care it should always be assessed whether the Anxiety Disorder diagnosis is incorrect, whether another comorbid disorder is the primary problem or whether there are exogenous factors like caffeine overuse, alcohol or substance use or medical diagnoses that contribute to treatment resistance (Fava et al., 2012; Roy‐Byrne, 2015). Also, in some studies it was not possible to assess whether previous treatment failures that were counted towards presence of TR‐AD consisted of evidence‐based antianxiety treatments. Finally, although psychological treatments like CBT were repeatedly proven effective in Anxiety Disorders (Bandelow et al., 2015; Carpenter et al., 2018), in many parts of the world they are not readily available (Saxena, Thornicroft, Knapp, & Whiteford, 2007). Therefore, generalizability of our findings may be limited in these regions.

Furthermore, for the purpose of this study we regarded TR‐AD, “refractory anxiety” and other related terms as synonyms. Even though this approach is in line with the majority of the studies, a minority consider TR‐AD and “refractory anxiety” to be different entities. For instance, in a Cochrane review, Ipser et al. (2006) propose the term TR for Anxiety Disorder patients who failed one pharmacologic treatment, whereas “refractory anxiety” refers to Anxiety Disorder patients with more than one failed treatment. Their approach can be viewed as a staging approach, distinguishing patients with end‐stage TR‐AD disorders from those with early stage TR‐AD. This approach is also advocated by Cosci and Fava (2013), who propose a staging model for TR Panic Disorders. In their model, the level of TR increases when more treatment regimens within pharmacologic, psychological and combination treatment have failed. In a number of treatment algorithms, a stepped care approach hints to the author's underlying assumption of a staging model for levels of TR (National Institute for Health & Clinical Excellence, 2011). In staging models, treatment decision making is based on the stage of disease progression in which the patient currently is classified. This could lead to evidence‐based stepped‐care treatment algorithms. We did not incorporate this staging paradigm for TR‐AD into the current paper, as no consensus exists for definitions of TR‐AD, nor for staging approaches in TR‐AD.

Future studies could empirically investigate the consensus definition for TR‐AD. A first step could be to apply the proposed TR‐AD definition to an Anxiety Disorder cohort and evaluate the longitudinal course of patients with TR‐AD compared to patients without TR‐AD. Possibly, this could also yield risk factors for development of TR‐AD. Further research could also focus on the validity of a staging approach in TR‐AD, as suggested by Cosci and Fava (2013) and Ipser et al. (2006).

In depression, a staging paradigm for TR is in use with the Maudsley Staging Method (Fekadu et al., 2009; Peeters et al., 2016; van Belkum et al., 2018). A similar approach could be beneficial for Anxiety Disorders. The criteria comprising TR‐AD that were described in the current paper could be studied on their merits as individual components in a staging method for TR‐AD, to reflect the various degrees of TR‐AD.

## 5 CONCLUSIONS

The majority of studies on treatment resistant Anxiety Disorders (TR‐AD) do not demarcate this phenomenon. Across studies that do provide a definition for TR‐AD there are many inconsistencies, which are likely to halt progress in Anxiety Disorder research. The current systematic review integrated the current literature into a consensus definition for TR‐AD (see Panel 2). This consensus definition should be regarded as a first step to advance the field further. The definition provided in this paper could contribute in harmonization of the process of evaluating presence of TR‐AD, which is a necessary first step towards improvement of the prognosis for TR‐AD patients.

## Supporting information

Supporting information
