# Supplementary material for: Aligning the many definitions of treatment resistance in anxiety disorders: A systematic review
Source: Depress Anxiety. 2019 Jun 23;36(9):801–12. doi: 10.1002/da.22895 (PMC6771798; doi:10.1002/da.22895)
Supplement: Supplementary file 1 — Supporting information [file DA-36-801-s001.docx]

**Supplementary materials**

**Abbreviations used in eTables**

| ACT Acceptance and Commitment Therapy | MGHAP CGI-S Massachusetts General Hospital Anchored Panic CGI-S |
| --- | --- |
| ADs antidepressants | MI Mobility Inventory |
| BDZ Benzodiazepine | *na* not available |
| CAS+PA Clinical Anxiety Scale with panic attacks | OCD Obsessive Compulsive Disorder |
| CAU care as usual | OQ-45.2 outcome measure 45.2 |
| CBT Cognitive Behavioral Therapy | PAS Panic and Agoraphobia Scale |
| CGI-I Clinical Global Impression Improvement Scale | PD Panic Disorder (with or without agoraphobia) |
| CGI-S Clinical Global Impression Severity Scale | PDSS Panic Disorder Severity Scale |
| DSM-IV Diagnostic and Statistical Manual, fourth edition | PSQ Panic Self Questionnaire |
| ECT Electroconvulsive therapy | PTSD Post Traumatic Stress Disorder |
| ER extended release | RCT randomized controlled trial |
| FU follow-up | refr refractory |
| GAD Generalized Anxiety Disorder | SAD Social Anxiety Disorder |
| GAF Global Assessment of Functioning | SCL-90-R Symptom Checklist-90, Revised |
| HAM-A Hamilton Anxiety Rating Scale | SDS Sheehan Disability Scale |
| HAM-D Hamilton Depression Rating Scale | SNRI selective Serotonin and Norepinephrine Reuptake Inhibitor |
| H high | SP Specific Phobia |
| IIP-64 Inventory of Interpersonal Problems | SRI Serotonin Reuptake Inhibitor |
| L low | SSRI Selective Serotonin Reuptake Inhibitor |
| LSAS Leibowitz Social Anxiety Scale | TCA Tricyclic Antidepressant |
| M medium | TR treatment resistant |
| MAO-I Monoamine Oxidase inhibitor | XR extended release |
| MDD Major Depressive Disorder |  |

**eTable 1. Study characteristics of included trials, cohort studies and meta-analyses.**

| Authors, year of publication | Study design | No of studies/ subjects | Population | Intervention | Comparator | Follow-up (FU) duration | Primary outcome | Conclusions |
| --- | --- | --- | --- | --- | --- | --- | --- | --- |
| Aarre, 2003 | Case series | n=7 | Refractory SAD | Phenelzine (15-90 mg twice daily) | None | unspecified | Anxiety severity | FU > baseline |
| Bakish et al., 1995 | Retrospective cohort | n=31 | Resistant PD, MDD, or Dysthymia | Moclobemide (35-800 mg/day) and SSRI (1-300 mg/day) combination treatment | None | unspecified | Symptom severity | FU > baseline |
| Barton, Karner, Salih, Baldwin, & Edwards, 2014 | Systematic review, meta-analysis | 0 studies included | TR GAD, PD, SAD, SP, OCD and PTSD in older adults | Any interventions for TR | Placebo, no intervention, or another active intervention | unspecified | Anxiety severity | no evidence base for treatments of TR anxiety in older adults |
| Brawman-Mintzer, Knapp, & Nietert, 2005 | RCT | n=40 | Refractory GAD | Adjunctive risperidone (0.5-1.5 mg/day) | Placebo | 5 weeks | Anxiety severity | Risperidone > placebo |
| Castle, Gray, Neehoff, & Glue, 2017 | Combined open-label-trial/ RCT | *N=18* | TR GAD and/or SAD | Ketamine in 0.25 mg/kg, 0.5 mg/kg, and 1 mg/kg | midazolam 0.01 mg/kg | 7 days | self-reported levels of dissociation | Intervention induces more dissociative symptoms |
| Cowley, Ha, & Roy-Byrne, 1997 | Retrospective cohort | n=106 | TR PD | No intervention | None | Not specified | Predictors of treatment failure | Predictors: Medication intolerance, inadequate dose or duration |
| De Salas-Cansado et al., 2013 | Cost-effectiveness study | n=902 | Refractory GAD | Pregabalin mono- or adjunctive therapy (average dose 218 mg/day) | CAU | 6 months | Cost-effectiveness | Intervention > CAU |
| Durham, Higgins, Chambers, Swan, & Dow, 2012 | Follow-up after retrospective cohort | n=336 | CBT study participants with GAD, PD, PTSD or MDD | Adjunctive CBT | None | 2-14 years post-treatment | Severity of anxiety, functional status, healthcare usage | 38% recovered with minimal treatment, 30% poor outcome despite treatment |
| Gabriel, 2010 | Open-label trial | n=32 | Refractory GAD | Adjunctive Adderall XR (5-50 mg/day) | None | 12 weeks | Anxiety severity | FU > baseline |
| Gabriel & Violato, 2011 | Open-label trial | n=29 | Refractory GAD | Adjunctive atomoxetine (10-40 mg twice daily) | None | 12 weeks | Anxiety severity | FU > baseline |
| George et al., 2008 | Open-label trial | n=11 | TR PD, OCD and PTSD | Vagus nerve stimulation | None | 4.5 years | Symptom severity | FU > baseline |
| Glue et al., 2017 | Open-label trial | *N=12* | TR GAD and/or SAD | Ketamine in 0.25 mg/kg, 0.5 mg/kg, and 1 mg/kg | none | 7 days | anxiety severity | Dose response relation present |
| Glue et al., 2018 | Open-label trial | *N=20* | TR GAD and/or SAD who responded in ketamine trial | Ketamine maintenance (3 months) | none | 3 months post intervention | - Anxiety severity  - tolerability  - social and work functioning | Maintenance treatment sustains remission |
| Gloster et al., 2015 | RCT | n=43 | TR PD | Adjunctive acceptance and commitment therapy (ACT) | Waiting-list | 6 months | Symptom severity | ACT > waiting-list |
| Heldt et al., 2003 | Open-label trial | n=32 | TR PD | Adjunctive CBT | None | 12 weeks | 1: Symptomatic change  2: negative outcome predictors | 1: FU > baseline  2: Predictors: depression, neurotic defense style |
| Heldt et al., 2006 | Open-label trial | n=64 | TR PD | Adjunctive CBT | None | 12 weeks + 1-year post-treatment | 1: Anxiety severity  2: Negative outcome predictors | 1: FU > baseline  2: Predictors: comorbid dysthymia, SP, GAD |
| Hirschmann et al., 2000 | RCT | n=26 | TR PD | Adjunctive pindolol (2.5 mg three times daily) | Placebo | 4 weeks | Anxiety severity | Intervention > placebo |
| Hoge et al., 2008 | Open-label trial | n=23 | Refractory GAD and PD | Adjunctive aripiprazole (2.5- 30 mg/day) | None | 8 weeks | Anxiety severity | FU > baseline |
| Hollifield, Thompson, Ruiz, & Uhlenhuth, 2005 | Open-label trial | n=10 | Refractory PD | Olanzapine (2.5-20 mg/day) after tapering off current medication | None | 8 weeks | Anxiety severity, impairment and safety | FU > baseline |
| Ipser et al., 2006 | Systematic review and meta-analysis | 28 RCTs | TR GAD, PD, SAD, OCD and PTSD | Pharmacotherapeutic augmentation | Placebo or other medication (e.g. monotherapy) | 4-20 weeks | Response | Intervention > control |
| Katzman et al., 2008 | Open-label trial | n=40 | TR GAD | Adjunctive quetiapine (25-800 mg/day) | None | 12 weeks | Anxiety severity | FU > baseline |
| Kinrys, Vasconcelos E Sa, & Nery, 2007 | Open-label trial | n=10 | Refractory GAD, PD, SAD and PTSD | Adjunctive zonisamide (100-300 mg/day) | None | 4-20 weeks | Anxiety severity | FU > baseline |
| Kinrys et al., 2007 | Retrospective cohort | n=40 | Refractory GAD, PD, SAD and PTSD | Adjunctive levetiracetam (250-3000 mg/day) | None | 9.3 ± 5.1 weeks | Anxiety severity | FU > baseline |
| Lohoff, Etemad, Mandos, Gallop, & Rickels, 2010 | RCT | n=62 | TR GAD | Adjunctive ziprasidone (20-80 mg/day) | Placebo | 8 weeks | Anxiety severity | Intervention = placebo |
| Menza, Dobkin, & Marin, 2007 | Open-label trial | n=9 | TR GAD | Adjunctive aripiprazole | None | 6 weeks | Anxiety severity and improvement | FU > baseline |
| Milrod et al., 2016 | Prospective cohort | n=46 | Refractory anxiety disorders | Adjunctive Panic Focused Psychodynamic Psychotherapy-eXtended Range | None | 12 weeks | 1: Symptomatic change after FU  2: Separation anxiety prevalence  3: Biomarker levels | 1: FU > baseline  2: 80% had separation anxiety  3: no significant change |
| Ociskova, Prasko, Latalova, Kamaradova, & Grambal, 2016 | Open-label trial | n=109 | TR GAD, PD, SP and mixed anxiety and depressive disorder | Inpatient CBT + pharmacotherapy | Inpatient psychodynamic therapy + pharmacotherapy | 6 weeks | Psychological predictors of treatment outcome | Multiple predictors found |
| Otto, Pollack, Penava, & Zucker, 1999 | Case series | n=24 | TR PD | Adjunctive CBT | None | 12 weeks | Anxiety severity | FU > baseline |
| Pallanti & Quercioli, 2006 | Open-label trial | n=29 | TR SAD | Escitalopram (10-20 mg/day) after wash-out | None | 12 weeks | Anxiety severity | FU > baseline |
| Patterson & Van Ameringen, 2016 | Systematic review and meta-analysis | 6 RCTs, n=557 | TR GAD, PD and SAD | Pharmacotherapeutic or CBT augmentation to SRI treatment | Placebo | 6-12 weeks | Clinical improvement | Intervention = placebo |
| Pollack, Otto, Kaspi, Hammerness, & Rosenbaum, 1994 | Open-label trial | n=15 | Refractory PD | Adjunctive CBT | None | 12 weeks + 1-8 months post-treatment | Anxiety severity | FU > baseline |
| Pollack et al., 2006 | RCT | n=21 | TR GAD | Adjunctive olanzapine (2.5-20 mg/day) | Placebo | 6 weeks | Anxiety severity | Intervention > placebo |
| Rickels et al., 2012 | RCT | n=356 | Refractory GAD | Adjunctive pregabalin (150-600 mg/day) | Adjunctive placebo | 8 weeks | Anxiety severity | Intervention > placebo |
| Simon et al., 2006 | Open-label trial | n=30 | Refractory GAD, PD and SAD | Adjunctive risperidone (0.25-3 mg/day) | None | 8 weeks | Anxiety severity | FU > baseline |
| Simon et al., 2009 | Combined RCT/ Open-label trial | n=46 | Refractory PD | Phase 1: Prospective assessment of refractoriness  Phase 2: SSRI dose optimization  Phase 3: Adjunctive CBT | Phase 1: No control arm  Phase 2: placebo  Phase 3: medication optimization | 24 weeks + 3 months post-treatment | Anxiety severity | Phase 2 and 3: Both groups improved, Intervention = placebo |
| Snyderman et al., 2005 | Open-label trial | n=13 | TR GAD | Ziprasidone (20-80 mg/day) | None | 7 weeks | Anxiety severity | FU > baseline |
| Solbakken & Abbass, 2015 | Open-label trial | n=60 | TR anxiety or depressive disorder | Intensive inpatient treatment program^1^ | None | 8 weeks + 14 months post-treatment | Symptom severity | FU > baseline |
| Solbakken & Abbass, 2016 | Open-label trial | n=95 | TR anxiety or depressive disorder | Intensive inpatient treatment program^1^ | None | 8 weeks + 14 months post-treatment | Symptom severity | FU > baseline |
| Tesar & Rosenbaum, 1986 | Case series | n=10 | TR PD | Adjunctive clonazepam (1.5-8 mg/day) | None | 8-68 weeks | Symptom improvement | FU > baseline |
| Worthington III, Kinrys, Wygant, & Pollack, 2005 | Open-label trial | n=17 | TR anxiety disorder or MDD | Adjunctive aripiprazole (7.5-30 mg/day) | None | 12 weeks | Symptom severity | FU > baseline |
| Yoshinaga et al., 2016 | Assessor blinded open-label trial | n=42 | TR SAD | Adjunctive CBT | CAU | 16 weeks | Anxiety severity | Intervention > CAU |
| Zoun et al., 2016 | Trial protocol | *na* | TR anxiety and depressive disorders | Self-management for Chronic Anxiety and Depression | CAU | 18 months | 1: Quality of life  2: Symptom severity  3: Costs | Not yet available |

^1^ the program consisted of individual psychotherapy, group psychotherapy, psychopharmacological treatment, and therapeutic group activities.

**eTable 2. Study characteristics for included reviews, treatment guidelines and book chapters.**

| Authors, year of publication | Study design | Population | Study description |
| --- | --- | --- | --- |
| Bakker, Van Balkom, & Stein, 2005 | Narrative review | Treatment refractory PD | Examines first-line pharmacotherapy, optimal duration of maintenance pharmacotherapy, and optimal approach to treatment refractoriness in PD patients. |
| Baldwin & Polkinghorn, 2005 | Book chapter | Non-responsive GAD | Examines first-line pharmacotherapy, optimal duration of treatment, and best interventions after non-response of first-line and second-line treatments in GAD patients |
| Bandelow, Zohar, Hollander, Kasper, & Moller, 2002 | Guideline | GAD, PD, SAD, SP, OCD and PTSD | Guideline for the pharmacological treatment of GAD, PD, SAD, SP, OCD and PTSD |
| Bandelow, 2008 | Guideline | GAD, PD, SAD, SP, OCD and PTSD | First revision of a guideline for the pharmacological treatment of GAD, PD, SAD, SP, OCD and PTSD |
| Bandelow et al., 2008 | Narrative review | GAD, PD, SAD and OCD | Summary of pharmacological treatment recommendations for GAD, PD, SAD and OCD |
| Bystritsky, 2006 | Narrative review | TR GAD, PD, SAD and OCD | Reviews reasons for TR and strategies for improving outcome in TR patients |
| Chen & Tsai, 2016 | Narrative review | TR PD | Presents definitions, risk factors, pathophysiology hypotheses and therapeutic strategies for TR PD |
| Cosci & Fava, 2013 | Systematic review | PD | Synthesizes 78 studies to describe the different models of staging currently known in clinical psychology and psychiatry (including a staging model for panic disorder) |
| Deligiannidis & Rothschild, 2010 | Book chapter | GAD, OCD, PTSD, bipolar disorder, depressive disorders | Reviews the evidence base for the use of antipsychotic medication in this population |
| Holt & Lydiard, 2007 | Narrative review | PD | Reviews the pathophysiology, existing and emerging treatment options and strategies for optimizing treatment response in PD and presents a diagnostic approach for unresponsive PD |
| Lorenz, Jackson, & Saitz, 2010 | Narrative review | TR GAD | Reviews the safety and efficacy of atypical antipsychotics as augmentation to pharmacotherapy in TR GAD patients |
| National Institute for Health and Clinical Excellence, 2011 | Guideline | GAD and PD | Treatment guideline for PD and GAD patients |
| Pollack, 2009 | Narrative review | GAD | Summarizes clinical and demographic characteristics and pharmacotherapeutic strategies for GAD |
| Samuel, Zimovetz, Gabriel, & Beard, 2011 | Systematic Review | GAD | Reviews eight studies with regard to efficacy and safety of treatments for refractory GAD |
| Starcevic, 2008 | Narrative review | PD | Reviews developments and future challenges in the treatment of PD |
| Stein et al., 2001 | Algorithm | SAD | Primary care pharmacotherapy algorithm for SAD |
| Stein et al., 2010 | Algorithm | SAD | Updated version of a primary care pharmacotherapy algorithm for SAD |
| Stein, 2003 | Algorithm | GAD, PD, SAD, OCD and PTSD | Pharmacotherapy algorithm for GAD, PD, SAD, OCD and PTSD |
| Stein, 2004 | Algorithm | GAD, PD, SAD, OCD, PTSD, MDD | Primary care pharmacotherapy algorithm for MDD, GAD, PD, SAD, PTSD and OCD |
| Van Ameringen, Mancini, & Patterson, 2009 | Book chapter | SAD and SP | Overview of efficacy of pharmacological treatments for SAD and SP |

**eTable 3. Definitions for treatment resistance in anxiety disorders, as used by authors.**

| Authors, year of publication | Population | Degree of precision | Treatment resistance definition |
| --- | --- | --- | --- |
| Aarre, 2003 | Refractory SAD | **Medium** | “failing to respond to other treatments that have been proved effective in this condition” |
| Bakish et al., 1995 | Resistant PD, MDD, or Dysthymia | **Medium** | “conventional treatment for their psychiatric disorder failed” |
| Bakker et al., 2005 | Treatment refractory PD | **Medium** | “not all patients respond to the first trial of medication” |
| Baldwin & Polkinghorn, 2005 | Non-responsive GAD | **Low** | “patients who have not responded to first-line or second-line treatments” |
| Bandelow et al., 2002 | GAD, PD, SAD, SP, OCD and PTSD | **Medium** | “When initial treatment fails”  PD: “patients (…) who were resistant to several antipanic drug treatments”  SAD: “partial response to a SSRI”, “non-responders to SSRIs” |
| Bandelow, 2008 | GAD, PD, SAD, SP, OCD and PTSD | **High** | “do not fulfill response criteria after initial standard treatment”, “While no universally accepted criteria exist, a commonly used threshold for response is a >50% improvement in the total score of a …) rating scale” |
| Bandelow et al., 2008 | GAD, PD, SAD and OCD | **High** | “(…) do not fulfill response criteria after initial standard treatment. While no universally accepted criteria exist, a commonly used threshold for response is a 50% improvement in the total score of a (…) rating scale”  PD: “When initial treatments have failed”, “patients having residual symptoms despite being on an adequate dose of medication”, “patients who responded insufficiently to CBT”  SAD: “non-responsive to standard treatment”  GAD: “patients remaining symptomatic despite initial anxiolytic treatment” |
| Barton et al., 2014 | TR GAD, PD, SAD, SP, OCD and PTSD in older adults | **High** | “no evidence of substantial improvement after 4 weeks’ treatment with a treatment for which there is evidence of clinical effectiveness in the treatment of anxiety” |
| Brawman-Mintzer et al., 2005 | Refractory GAD | **Medium** | “patients who remain symptomatic despite ongoing standard anxiolytic treatment” |
| Bystritsky, 2006 | TR GAD, PD, SAD and OCD | **High** | “no restoration or near restoration of functional status in the presence (absence) of tolerable treatment” |
| Castle et al., 2017 | TR GAD and/or SAD | **Medium** | “HAM-A^1^ score of >20, and/or a (…) LSAS^1^ score of >60“ |
| Chen & Tsai, 2016 | TR PD | **High** | “the failure to achieve either of two remission criteria after at least 6 months of optimal treatment”  Remission criteria 1: complete resolution in 5 principal domains: panic attacks (…), anticipatory anxiety, panic-related phobias, well-being/severity of illness, and functional and social impairment caused by the panic disorder  Remission criteria 2: (…) PDSS ≤ 3, HAM-D ≤ 7 |
| Cosci & Fava, 2013 | PD | **High** | Stage 1: a pharmacological/psychological intervention fails to give benefits  Stage 2: the failure involves 2 different interventions, including at least 1 involving psychotherapeutic treatment  Stage 3: involves the failure of 3 or more adequate therapeutic interventions, including at least 1 involving Psychotherapy  Stage 4: there is failure of 3 or more adequate therapeutic trials, including at least 1 combination of psychotherapy and pharmacotherapy.” |
| Cowley et al., 1997 | TR PD | **Medium** | TR: “patients [who] fail initial treatment”  True TR: “failure to respond to an effective medication given at a therapeutic dose for an appropriate length of time” |
| Deligiannidis & Rothschild, 2010 | GAD, OCD, PTSD, bipolar disorder, depressive disorders | **Medium** | “partial and non-responders to the first-line GAD therapies” |
| De Salas-Cansado et al., 2013 | Refractory GAD | **High** | “persistent symptoms/suboptimal response (…) after a standard dose regimen of any anti-anxiety drug, alone or in combination, for at least 6 months” |
| Durham et al., 2012 | CBT study participants with GAD, PD, PTSD or MDD | **Medium** | “a clinical diagnosis of at least one anxiety disorder despite receiving either a moderate amount or a lot of interim treatment”, “with either medication or psychological therapy” |
| Gabriel, 2010 | Refractory GAD | **Medium** | “failed to respond to at least one 8-week trial of SSRI, or SNRI (…) with > 50% reduction in anxiety symptoms from baseline” |
| Gabriel & Violato, 2011 | Refractory GAD | **Medium** | “partial response to at least one 8-week trial of SSRI or SNRI” |
| George et al., 2008 | TR PD, OCD and PTSD | **High** | “tried and failed pharmacotherapy and psychotherapy” |
| Glue et al., 2017 | TR GAD and/or SAD | **Medium** | “(…) HAM-A score of >20, and/or a (…) LSAS score of >60“ |
| Glue et al., 2018 | TR GAD and/or SAD who responded in ketamine trial | **Medium** | “Hamilton Anxiety Scale (…) score of >20, and/or a Liebowitz Social Anxiety Scale (…) score of >60“ |
| Gloster et al., 2015 | TR PD | **Medium** | “have had one or more previous courses of psychological and/or pharmacological treatment consistent with state-of-the-art practice” |
| Heldt et al., 2003 | TR PD | **Medium** | “the patients must have had residual symptoms of panic attacks, anticipatory anxiety and phobic avoidance despite having been on an adequate and stable dose of an SSRI for at least 4 months” |
| Heldt et al., 2006 | TR PD | **Medium** | “patients had to have residual symptoms of PD such as panic attacks, anticipatory anxiety and phobic avoidance despite being on a stable dose of medications for at least 4 months” |
| Hirschmann et al., 2000 | TR PD | **Medium** | “less than 20% reduction in score (…) after an 8-week trial of fluoxetine 20mg/day” |
| Hoge et al., 2008 | Refractory GAD and PD | **Medium** | “DSM-IV criteria for GAD or PD despite initial pharmacotherapy with an adequate (or highest tolerated) dose of an anxiolytic agent, including an SSRI or a (…) SNRI and/or BDZs or trazodone or bupropion, initiated at least 8 weeks prior to study initiation” |
| Hollifield et al., 2005 | Refractory PD | **High** | “having failed two adequate therapeutic trials of either medication or (…) CBT” |
| Holt & Lydiard, 2007 | PD | **High** | “an inadequate response to what is generally considered adequate treatment” |
| Ipser et al., 2006 | TR GAD, PD, SAD, OCD and PTSD | **High** | TR: “fail to respond to a first-line intervention” treatment refractory: “no change or whose symptoms worsen following several different interventions will be referred to as treatment-refractory” |
| Katzman et al., 2008 | TR GAD | **Medium** | “not responding to (…) a traditional therapy (…) a minimum of 8 weeks’ treatment with an appropriate dose of a traditional anxiolytic” |
| Kinrys et al., 2007 | Refractory GAD, PD, SAD and PTSD | **Medium** | “history of persistent anxiety despite adequate anxiolytic treatment (defined as 8 weeks or more of treatment (…)” |
| Kinrys et al., 2007 | Refractory GAD, PD, SAD and PTSD | **Medium** | “did not have a full response during their current episode despite an adequate dose and duration of their anxiolytic trial, defined as persistence of anxiety symptoms (…)” |
| Lohoff et al., 2010 | TR GAD | **Medium** | “treatment failure of at least 1 adequate trial of an SSRI, an SNRI, a BDZ, or a combination of these agents” |
| Lorenz et al., 2010 | TR GAD | **High** | “not respond to at least one antidepressant at an adequate dose for an adequate duration” |
| Menza et al., 2007 | TR GAD | **Medium** | “a (…) HAM-A score of 14 or greater, and a (…) CGI-S score of at least 4 (moderately ill). (…) patients had been treated with an antidepressant at a therapeutic dose (fluoxetine, 40 mg; paroxetine, 40 mg; escitalopram, 20 mg; bupropion, 250mg; venlafaxine, 150 mg; and mirtazapine, 30 mg) for an adequate duration (at least 6 weeks).” |
| Milrod et al., 2016 | Refractory anxiety disorders | **Medium** | “prominent anxiety (…) and could provide evidence of nonresponse (clinically persistent anxiety) to at least one evidence-based antianxiety treatment, specifically: (a) at least 2 months of an adequately dosed SSRI or TCA equivalent (…) or (b) ≥11 weeks of CBT” |
| National Institute for Health and Clinical Excellence, 2011 | GAD and PD | **Medium** | “severe anxiety with marked functional impairment in conjunction with: a risk of self-harm or suicide or significant comorbidity, such as substance misuse, personality disorder or complex physical health problems or self-neglect or an inadequate response to step 3 interventions.” |
| Ociskova et al., 2016 | TR GAD, PD, SP and mixed anxiety and depressive disorder | **Medium** | “patients were resistant to outpatient pharmacological and psychotherapeutic treatment (i.e., they were chronically unresponsive to standard outpatient pharmacological and/or psychological treatment)” |
| Otto et al., 1999 | TR PD | **Medium** | “Current CGI-S ≥ 4 with a minimum of 2 months of treatment with any of the following (…) (1) Any tricyclic (…) (2) Fluoxetine (…) (3) Sertraline (…) (4) Paroxetine (…) (5) Nefazodone (…) (6) Phenelzine (…) (7) Alprazolam (…) (8) Clonazepam (…)” |
| Pallanti & Quercioli, 2006 | TR SAD | **Medium** | “failed at least one adequate trial of paroxetine treatment (…)  ‘Failure’ was defined as:  1: experiencing less than a 35% decrease from baseline (…), and a score of 'minimal improvement' or less on the (…) CGI-I after 12 weeks of treatment  2: having stopped the medication in the first 3 weeks of treatment because of intolerable side effects or lack of compliance” |
| Patterson & Van Ameringen, 2016 | TR GAD, PD and SAD | **Medium** | “either a less than 50% improvement in the total score of a commonly used anxiety rating scale or a nonresponse to an adequate dose of first-line pharmacological treatment of a SRIs for 4–5 weeks.” |
| Pollack et al., 1994 | Refractory PD | **Medium** | Treatment refractory: “incomplete response to an adequate trial of medication”  Incomplete response to pharmacotherapy: “the continued presence, despite at least 3 months of medication treatment, of panic attacks, phobic fear or avoidance, or anticipatory anxiety severe enough to interfere with function.“ |
| Pollack et al., 2006 | TR GAD | **Medium** | “remain symptomatic despite treatment with standard agents such as the SSRIs” |
| Pollack, 2009 | GAD | **Medium** | “remaining symptomatic or to not respond at all to first-line medication” |
| Rickels et al., 2012 | Refractory GAD | **Medium** | “partial response to 8 weeks of (…) treatment with venlafaxine-extended release (XR), escitalopram, or paroxetine, and who had a suboptimal response (by history) to at least one previous treatment for GAD.” |
| Samuel et al., 2011 | GAD | **Medium** | “failed to respond adequately to at least one earlier treatment for GAD.” |
| Simon et al., 2006 | Refractory GAD, PD and SAD | **Medium** | “significant persistent symptoms despite initial pharmacotherapy (…) an adequate (or maximally tolerated) dose of an established anxiolytic (SSRI or SNRI and/or (…) BDZs) initiated at least 8 weeks prior” |
| Simon et al., 2009 | Refractory PD | **Medium** | “lack of remission” |
| Snyderman et al., 2005 | TR GAD | **Medium** | “a score of 4 or greater on the (…) CGI-S, a (…) HAM-A score 16 or greater after 8 weeks of treatment with at least 1 first-line antianxiety agent” |
| Solbakken & Abbass, 2015 | TR anxiety or depressive disorder | **Medium** | “failure to respond with symptomatic relief and improved occupational or interpersonal functioning to three or more prior attempts at treatment for the ongoing psychiatric disorder. The previous treatment attempts could be either medication efforts or psychotherapeutic/psychosocial efforts, or most commonly a combination of both” |
| Solbakken & Abbass, 2016 | TR anxiety or depressive disorder | **Medium** | “failure to respond with symptomatic relief and improved occupational or interpersonal functioning despite three or more prior attempts at treatment for the ongoing disorder. The previous treatment attempts could be either medication efforts or psychotherapeutic/psychosocial efforts, or most commonly a combination of both” |
| Starcevic, 2008 | PD | **High** | “a patient with PD can be considered resistant to pharmacological treatment if he/she has not fully responded to an adequate treatment with two SSRIs, venlafaxine ER, one of the TCAs (imipramine or clomipramine), a high-potency BDZs, or a combination of one of these antidepressants with a BDZ” |
| Stein et al., 2001 | SAD | **Low** | “who have failed to respond to a (…) SSRI” |
| Stein et al., 2010 | SAD | **Medium** | “fail to respond to treatment with the first SSRI/SNRI” |
| Stein, 2003 | GAD, PD, SAD, OCD and PTSD | **Medium** | “failure of an adequate clinical trial of medication” |
| Stein, 2004 | GAD, PD, SAD, OCD, PTSD, MDD | **Low** | “failure of an adequate clinical trial of medication” |
| Tesar & Rosenbaum, 1986 | TR PD | **Low** | “failure to respond to, or refusal to be treated with, each of the major antipanic agents, including a TCA, a MAO-i, and alprazolam. Failure to respond is defined by presence of one or more of the following: a) lack of therapeutic effect, b) tolerance to drug effect, c) suboptimal therapeutic effect, or d) medication intolerance due to side effects” |
| Van Ameringen et al., 2009 | SAD and SP | **Low** | “not respond, or has a partial response to a standard, first line treatment” |
| Worthington III et al., 2005 | TR anxiety disorder or MDD | **Medium** | “did not have a full response during their current episode despite an adequate dose and duration of their SSRI trial” |
| Yoshinaga et al., 2016 | TR SAD | **Medium** | “ ‘patients who remain symptomatic following antidepressant treatment’ ” |
| Zoun et al., 2016 | TR anxiety and depressive disorders | **Medium** | “prolonged treatment in a specialized outpatient mental health service according to the professional is unlikely to improve clinical outcomes (e.g. achieve remission)”, “two years in specialized mental health care; received at least one psychological treatment and at least three medication steps according to the national multidisciplinary guidelines on anxiety and depressive disorders” |

^1^ Commonly used measurement instruments, diagnoses, treatment modalities (e.g.: SSRIs, CBT) were abbreviated in this table even if the original paper did not abbreviate them.

**eTable 4. Definitions for treatment resistance in anxiety disorders, per included study and per criterion.**

|  |  | Psychotherapy | | Psychopharmacological treatment | | |  |  |  |  |  |  |  |
| --- | --- | --- | --- | --- | --- | --- | --- | --- | --- | --- | --- | --- | --- |
| Authors, year | Minimal number of treatments | Unspecified^1^ | CBT | Unspe-cified^1^ | SSRIs and/or SNRIs | other | Other biological treatments | Minimal length of treatment | Treatment response criterion | Duration of disorder | Severity of symptoms | Functional impair-ment | Comorbidity |
| Aarre, 2003 | x | v |  | v |  |  | x | x | CGI-I ≤2 | x | x | x | x |
| Bakish et al., 1995 | 2 | x | x |  | v | v | x | 4 weeks | x | x | x | x | x |
| Bakker et al., 2005 | 1 | x | x |  | v | x | x | 4 weeks | x | x | x | x | x |
| Baldwin & Polkinghorn, 2005 | x | x | x | v |  |  | x | x | x | x | x | x | x |
| Bandelow et al., 2002 | 1 | x | x | v |  |  | x | 4-6 weeks | x | x | x | x | x |
| Bandelow, 2008 | 1 | x | x | v |  |  | x | 4-6 weeks | ∆HAM-A ≥50% | x | x | x | x |
| Bandelow et al., 2008 | 1 | x | x | v |  |  | x | 4-6 weeks | ∆HAM-A ≥50% | x | x | x | x |
| Barton et al., 2014 | 1 | v |  | v |  |  | x | 4 weeks | ∆anxiety scale ≥50% | x | x | x | x |
| Brawman-Mintzer et al., 2005 | 1 | x | x |  | v | v | x | 4 weeks | x | x | HAM-A ≥18 (items 1&2 ≥2), Covi Anxiety Scale: moderate, CGI-S ≥4 | x | x |
| Bystritsky, 2006 | x | v |  | v |  |  | x | x | x | x | x | v | x |
| Castle et al., 2017 | x | x | x | x | x | x | x | x | x | x | HAMA≥20 LSAS≥60 | x | x |
| Chen & Tsai, 2016 | 1 | x | x | x | x | x | x | 6 months | 1: No panic attacks, ≤mild avoidance, HAM-A ≤7-10, SDS ≤1 on each item, HAM-D ≤7, or 2: PDSS ≤3 and no item >1 | x | 1: HAM-A >7-10, HAM-D >7, or, 2: PDSS >3, or any item >1 | 1: SDS >1 on each item, or 2: x | 1: x, or 2: HAM-D ≤7 |
| Cosci & Fava, 2013 | from 1 to over 3 | from one trial (stage 1) to at least three trials including combination treatment (stage 4) | | | | | x | x | x | x | x | x | x |
| Cowley et al., 1997 | 1 | x | x | v |  |  | x | x | x | x | x | x | x |
| Deligiannidis & Rothschild, 2010 | x |  | v |  | v | v | x | x | x | x | x | x | x |
| De Salas-Cansado et al., 2013 | 1 | x | x | v |  |  | x | 6 months | ∆HAM-A ≥50%, HAM-A ≤9 | x | HAM-A ≥16 & CGI-S ≥3 | x | x |
| Durham et al., 2012 | “extensive treatment” | v |  | v |  |  | x | x | x | x | x | x | x |
| Gabriel, 2010 | 1 | x | x |  | v | x | x | 8 weeks | HAM-A <7 | x | HAM-A >7 & ∆HAM-A ≥50% | x | x |
| Gabriel & Violato, 2011 | 1 | x | x |  | v | x | x | 8 weeks | HAM-A <7 | x | HAM-A >10 | x | x |
| George et al., 2008 | 4 |  | v | v |  |  | x | x | ∆HAM-A ≥50% | x | HAM-A ≥20, CGI-S ≥4 | x | x |
| Glue et al., 2017 | x | x | x |  | x |  | x | x | x | x | HAMA≥20 LSAS≥60 | x | x |
| Glue et al., 2018 | x | x | x |  | x |  | x | x | x | x | HAMA≥20 LSAS≥60 | x | x |
| Gloster et al., 2015 | 1 |  | v | v |  |  | x | CBT: 20 sessions | PAS ≤18, CGI-S ≤3 | x | MI ≥1.5, CGI-S ≥4 | x | x |
| Heldt et al., 2003 | 1 | x | x |  | v | x | x | 4 months | No panic attacks, CGI≤2, ∆HAM-A, ∆agoraphobia, ∆anticipatory anxiety ≥50% | x | x | x | x |
| Heldt et al., 2006 | 1 | x | x | v |  |  | x | 4 months | No panic attacks, CGI≤2, ∆HAM-A, ∆agoraphobia, ∆anticipatory anxiety ≥50% | x | CGI-S ≥3 | x | x |
| Hirschmann et al., 2000 | 3 | x | x | v | v | x | x | 8 weeks | x | x | ∆PSQ & CAS+PA ≥20% | x | x |
| Hoge et al., 2008 | 1 | x | x |  | v | v | x | 8 weeks | x | x | GAD: HAM-A ≥16,  CGI-S ≥4;  PD: MGHAP CGI-S ≥4 | x | x |
| Hollifield et al., 2005 | 2 |  | v | v |  |  | x | 8 weeks | x | x | x | x | x |
| Holt & Lydiard, 2007 | 1 |  | v | v |  |  | x | x | x | x | x | x | x |
| Ipser et al., 2006 | TR: 1  refr: 2 | TR: x  refr: v |  | refr: v | TR: v | x | x | x | used a variety | x | x | x | x |
| Katzman et al., 2008 | 1 | x | x | v |  |  | x | 8 weeks | HAM-A ≤10 | x | CGI-S ≥5, HAM-A ≥20, >2 on anxious mood and tension | x | x |
| Kinrys et al., 2007 | 1 | x | x | v |  |  | x | x | CGI-I ≤2 | x | CGI-S ≥4 | x | x |
| Kinrys et al., 2007 | 1 | x | x |  | v | v | x | 8 weeks | CGI-I ≤2 | x | CGI-S ≥4 | x | x |
| Lohoff et al., 2010 | 1 | x | x |  | v | v | x | x | x | x | HAM-A ≥16, CGI-S ≥4 | x | x |
| Lorenz et al., 2010 | 1 | x | x | v |  |  | x | x | x | x | x | x | x |
| Menza et al., 2007 | x | x | x | v |  |  | x | 6 weeks | ∆HAM-A ≥50% | x | HAM-A ≥14, CGI-S ≥4 | x | x |
| Milrod et al., 2016 | 1 |  | v |  | v | v | x | 2 months (ADs), 11 weeks (CBT) | x | x | HAM-A >15 | x | x |
| National Institute for Health and Clinical Excellence, 2011 | 3 steps (in stepped care algorithm) |  | v |  | v | v | x | x | x | x | “severe” | “marked” | x |
| Ociskova et al., 2016 | x | v |  | v |  |  | x | x | CGI-S ≤2 | x | x | x | x |
| Otto et al., 1999 | 1 | x | x |  | v | v | x | 2 months | CGI-S ≤2 | x | CGI-S ≥4 | x | x |
| Pallanti & Quercioli, 2006 | 1 | x | x |  | v | x | x | 12 weeks | x | 1 year | ∆LSAS <35%, CGI-I ≥3 | x | x |
| Patterson & Van Ameringen, 2016 | 1 | x | x |  | v | x | x | 4-5 weeks | CGI-S ≤2 | x | SAD: ∆LSAS <50%, GAD/PD: ∆HAM-A <50%, PD: ∆PDSS <50% | x | x |
| Pollack et al., 1994 | 1 | x | x |  | v | v | x | 12 weeks | x | x | x | x | x |
| Pollack et al., 2006 | 1 | x | x |  | v | x | x | 6 weeks | ∆HAM-A ≥50%, HAM-A ≤7, CGI-S ≤2 | x | CGI-S ≥4, ∆HAM-A <50% | x | x |
| Pollack, 2009 | 1 | x | x |  | v | v | x | x | x | x | x | x | x |
| Rickels et al., 2012 | 2 | x | x |  | v | x | x | 8 weeks | ∆HAM-A ≥50% | x | HAM-A >16 | x | x |
| Samuel et al., 2011 | 1 | x | x | v |  |  | x | x | x | x | x | x | x |
| Simon et al., 2006 | 1 | x | x |  | v | v | x | 8 weeks | x | x | GAD: HAM-A ≥16, SAD: LSAS ≥70, PD: Panic CGI-S ≥4, All: CGI-S ≥4 | x | x |
| Simon et al., 2009 | 1 | x | x |  | v | x | x | 6 weeks | CGI-S ≤2, no panic attacks last week | x | CGI-S ≥3, ≥1 panic attack last week | x | x |
| Snyderman et al., 2005 | 1 | x | x | v |  |  | x | 8 weeks | ∆HAM-A ≥50%, HAM-A ≤7, CGI-I ≤2 | x | CGI-S ≥4, HAM-A ≥16 | x | x |
| Solbakken & Abbass, 2015 | 3 | v |  | v |  |  | x | x | OQ-45.2 ≤63, ∆OQ-45.2 ≥14, SCL-90-R ≤0.87, ∆SCL-90-R ≥0.32, IIP-64 ≤1.37, ∆IIP-64 ≥0.25 | x | x | “loss of function in multiple domains” | x |
| Solbakken & Abbass, 2016 | 3 | v |  | v |  |  | x | x | x | x | x | “loss of function in multiple domains” | x |
| Starcevic, 2008 | 1 or 2 | x | x |  | 2 SSRIs | v | x | x | x | x | x | x | x |
| Stein et al., 2001 | 1 | x | x |  | v | x | x | 8-12 weeks | x | x | x | x | x |
| Stein et al., 2010 | 1 | x | x |  | v | x | x | x | x | x | x | x | x |
| Stein, 2003 | 1 | x | x | v |  |  | x | PD: 8 weeks, SAD: 12 weeks | x | x | x | x | x |
| Stein, 2004 | 1 | x | x | v |  |  | x | 8 weeks | x | x | x | x | x |
| Tesar & Rosenbaum, 1986 | 3 | x | x |  | x | v | x | x | x | x | x | x | x |
| Van Ameringen et al., 2009 | 1 | x | x |  | v | x | x | x | x | x | x | x | x |
| Worthington III et al., 2005 | 1 | x | x |  | v | x | x | x | x | x | x | x | x |
| Yoshinaga et al., 2016 | 1 | v |  |  | v | x | x | 12 weeks | ∆LSAS ≥31% | x | LSAS ≥50, CGI-I ≥3 | x | x |
| Zoun et al., 2016 | 4 | v |  | v |  |  | x | x | x | 2 years | x | x | x |

**^1^** used when authors did not mention which specific type of intervention was mandatory in their definition of treatment resistance, for instance “psychotherapeutic treatment”, or “antidepressants”.
x= not included in definition, v= included in definition.

**References**

Aarre, T. F. (2003). Phenelzine efficacy in refractory social anxiety disorder: A case series. *Nordic Journal of Psychiatry*, *57*(4), 313–315. https://doi.org/10.1080/08039480310002110

Bakish, D., Hooper, C. L., West, D. L., Miller, C., Blanchard, A., & Bashir, F. (1995). Moclobemide and specific serotonin re-uptake inhibitor combination treatment of resistant anxiety and depressive disorders. *Human Psychopharmacology: Clinical and Experimental*, *10*(2), 105–109. https://doi.org/10.1002/hup.470100205

Bakker, A., Van Balkom, A. J. L. M., & Stein, D. J. (2005). Evidence-based pharmacotherapy of panic disorder. *International Journal of Neuropsychopharmacology*, *8*(3), 473–482. https://doi.org/10.1017/S1461145705005201

Baldwin, D. S., & Polkinghorn, C. (2005). Evidence-based pharmacotherapy of generalized anxiety disorder. In D. J. Stein, B. Lerer, S. Stahl, D. J. Stein (Ed), B. Lerer (Ed), & S. Stahl (Ed) (Eds.), *Evidence-based psychopharmacology.* (pp. 88–104). New York, NY, US: Cambridge University Press. Retrieved from http://search.ebscohost.com/login.aspx?direct=true&db=psyh&AN=2005-14567-004&site=ehost-live

Bandelow, B. (2008). The medical treatment of obsessive-compulsive disorder and anxiety. *CNS Spectrums*, *13*(9, 14), 37–47. Retrieved from http://www.embase.com/search/results?subaction=viewrecord&from=export&id=L352617762

Bandelow, B., Zohar, J., Hollander, E., Kasper, S., & Moller, H.-J. (2002). World Federation of Societies of Biological Psychiatry (WfSBP) Guidelines for the Pharmacological Treatment of Anxiety, Obsessive-Compulsive and Psttraumatic Stress Disorders. *World J Biol Psychiatry*, *3*(904758812), 171–199. https://doi.org/10.1080/15622970802465807

Bandelow, B., Zohar, J., Hollander, E., Kasper, S., Möller, H.-J., & WFSBP Task force on treatment guidelines for anxiety obsessive-compulsive post-traumatic stress disorders. (2008). World Federation of Societies of Biological Psychiatry (WFSBP) guidelines for the pharmacological treatment of anxiety, obsessive-compulsive and post-traumatic stress disorders - First revision. *World Journal of Biological Psychiatry*, *9*(4), 248–312. https://doi.org/10.1080/15622970802465807

Barton, S., Karner, C., Salih, F., Baldwin, D. S., & Edwards, S. J. (2014). Clinical effectiveness of interventions for treatment-resistant anxiety in older people: A systematic review. *Health Technology Assessment*, *18*(50), 1–62. https://doi.org/10.3310/hta18500

Brawman-Mintzer, O., Knapp, R. G., & Nietert, P. J. (2005). Adjunctive risperidone in generalized anxiety disorder: A double-blind, placebo-controlled study. *Journal of Clinical Psychiatry*, *66*(10), 1321–1325. Retrieved from http://www.embase.com/search/results?subaction=viewrecord&from=export&id=L41546513

Bystritsky, A. (2006). Treatment-resistant anxiety disorders. *Molecular Psychiatry*, *11*(9), 805–814. https://doi.org/10.1038/sj.mp.4001852

Castle, C., Gray, A., Neehoff, S., & Glue, P. (2017). Effect of ketamine dose on self-rated dissociation in patients with treatment refractory anxiety disorders. *Journal of Psychopharmacology*, *31*(10), 1306–1311. https://doi.org/10.1177/0269881117725685

Chen, M.-H., & Tsai, S.-J. (2016). Treatment-resistant panic disorder: clinical significance, concept and management. *Progress in Neuro-Psychopharmacology and Biological Psychiatry*, *70*, 219–226. https://doi.org/10.1016/j.pnpbp.2016.02.001

Cosci, F., & Fava, G. A. (2013). Staging of mental disorders: systematic review. *Psychotherapy and Psychosomatics*, *82*(1), 20–34. https://doi.org/10.1159/000342243

Cowley, D. S., Ha, E. H., & Roy-Byrne, P. P. (1997). Determinants of pharmacologic treatment failure in panic disorder. *Journal of Clinical Psychiatry*, *58*(12), 555–561. Retrieved from http://www.embase.com/search/results?subaction=viewrecord&from=export&id=L28100280

De Salas-Cansado, M., Álvarez, E., Olivares, J. M., Carrasco, J. L., Ferro, M. B., & Rejas, J. (2013). Modelling the cost-effectiveness of pregabalin versus usual care in daily practice in the treatment of refractory generalised anxiety disorder in Spain. *Social Psychiatry and Psychiatric Epidemiology*, *48*(6), 985–996. https://doi.org/10.1007/s00127-012-0606-6

Deligiannidis, K. M., & Rothschild, A. J. (2010). Mood and anxiety disorders. In A. J. Rothschild (Ed.), *The evidence-based guide to antipsychotic medications.* (1st ed., pp. 45–100). Arlington, VA, US: American Psychiatric Publishing, Inc. Retrieved from http://search.ebscohost.com/login.aspx?direct=true&db=psyh&AN=2010-03392-003&site=ehost-live

Durham, R. C., Higgins, C., Chambers, J. A., Swan, J. S., & Dow, M. G. T. (2012). Long-term outcome of eight clinical trials of CBT for anxiety disorders: Symptom profile of sustained recovery and treatment-resistant groups. *Journal of Affective Disorders*, *136*(3), 875–881. https://doi.org/10.1016/j.jad.2011.09.017

Gabriel, A. (2010). The mixed amphetamine salt extended release (Adderall XR, Max-XR) as an adjunctive to SSRIs or SNRIs in the treatment of adult ADHD patients with comorbid partially responsive generalized anxiety: an open-label study. *ADHD Attention Deficit and Hyperactivity Disorders*, *2*(2), 87–92. https://doi.org/10.1007/s12402-010-0025-z

Gabriel, A., & Violato, C. (2011). Adjunctive atomoxetine to SSRIs or SNRIs in the treatment of adult ADHD patients with comorbid partially responsive generalized anxiety (GA): An open-label study. *ADHD Attention Deficit and Hyperactivity Disorders*, *3*(4), 319–326. https://doi.org/10.1007/s12402-011-0063-1

George, M. S., Ward, H. E., Ninan, P. T., Pollack, M., Nahas, Z., Anderson, B., … Ballenger, J. C. (2008). A pilot study of vagus nerve stimulation (VNS) for treatment-resistant anxiety disorders. *Brain Stimulation*, *1*(2), 112–121. https://doi.org/10.1016/j.brs.2008.02.001

Gloster, A. T., Sonntag, R., Hoyer, J., Meyer, A. H., Heinze, S., Ströhle, A., … Wittchen, H.-U. (2015). Treating treatment-resistant patients with panic disorder and agoraphobia using psychotherapy: A randomized controlled switching trial. *Psychotherapy and Psychosomatics*, *84*(2), 100–109. https://doi.org/10.1159/000370162

Glue, P., Medlicott, N. J., Harland, S., Neehoff, S., Anderson-Fahey, B., Le Nedelec, M., … McNaughton, N. (2017). Ketamine’s dose-related effects on anxiety symptoms in patients with treatment refractory anxiety disorders. *Journal of Psychopharmacology*, *31*(10), 1302–1305. https://doi.org/10.1177/0269881117705089

Glue, P., Neehoff, S. M., Medlicott, N. J., Gray, A., Kibby, G., & McNaughton, N. (2018). Safety and efficacy of maintenance ketamine treatment in patients with treatment-refractory generalised anxiety and social anxiety disorders. *Journal of Psychopharmacology*, 269881118762073. https://doi.org/10.1177/0269881118762073

Heldt, E., Manfro, G. G., Kipper, L., Blaya, C., Isolan, L., & Otto, M. W. (2006). One-year follow-up of pharmacotherapy-resistant patients with panic disorder treated with cognitive-behavior therapy: Outcome and predictors of remission. *Behaviour Research and Therapy*, *44*(5), 657–665. https://doi.org/10.1016/j.brat.2005.05.003

Heldt, E., Manfro, G. G., Kipper, L., Blaya, C., Maltz, S., Isolan, L., … Otto, M. W. (2003). Treating Medication-Resistant Panic Disorder: Predictors and Outcome of Cognitive-Behavior Therapy in a Brazilian Public Hospital. *Psychotherapy and Psychosomatics*, *72*(1), 43–48. https://doi.org/10.1159/000067188

Hirschmann, S., Dannon, P. N., Iancu, I., Dolberg, O. T., Zohar, J., & Grunhaus, L. (2000). Pindolol Augmentation in Patients With Treatment-Resistant Panic Disorder: A Double-Blind, Placebo-Controlled Trial. *Journal of Clinical Psychopharmacology*, *20*(5), 556–559. https://doi.org/10.1097/00004714-200010000-00011

Hoge, E. A., Worthington III, J. J., Kaufman, R. E., Delong, H. R., Pollack, M. H., Simon, N. M., … Simon, N. M. (2008). Aripiprazole as augmentation treatment for refractory generalized anxiety disorder and panic disorder. *CNS Spectrums*, *13*(6), 522–527. Retrieved from http://www.embase.com/search/results?subaction=viewrecord&from=export&id=L351960777

Hollifield, M., Thompson, P. M., Ruiz, J. E., & Uhlenhuth, E. H. (2005). Potential effectiveness and safety of olanzapine in refractory panic disorder. *Depression and Anxiety*, *21*(1), 33–40. https://doi.org/10.1002/da.20050

Holt, R. L., & Lydiard, R. B. (2007). Management of treatment-resistant panic disorder. *Psychiatry*, *4*(10), 48–59. Retrieved from http://search.ebscohost.com/login.aspx?direct=true&db=psyh&AN=2007-16431-006&site=ehost-live

Ipser, J. C., Carey, P., Dhansay, Y., Fakier, N., Seedat, S., & Stein, D. J. (2006). Pharmacotherapy augmentation strategies in treatment-resistant anxiety disorders (Review). *Cochrane Database of Systematic Reviews*, (4). https://doi.org/10.1002/14651858.CD005473.pub2

Katzman, M. A., Vermani, M., Jacobs, L., Marcus, M., Kong, B., Lessard, S., … Gendron, A. (2008). Quetiapine as an adjunctive pharmacotherapy for the treatment of non-remitting generalized anxiety disorder: A flexible-dose, open-label pilot trial. *Journal of Anxiety Disorders*, *22*(8), 1480–1486. https://doi.org/10.1016/j.janxdis.2008.03.002

Kinrys, G., Vasconcelos E Sa, D., & Nery, F. (2007). Adjunctive zonisamide for treatment refractory anxiety. *International Journal of Clinical Practice*, *61*(6), 1050–1053. https://doi.org/10.1111/j.1742-1241.2007.01365.x

Kinrys, G., Worthington, J. J., Wygant, L., Nery, F., Reese, H., & Pollack, M. H. (2007). Levetiracetam as adjunctive therapy for refractory anxiety disorders. *Journal of Clinical Psychiatry*, *68*(7), 1010–1013. Retrieved from http://www.embase.com/search/results?subaction=viewrecord&from=export&id=L47340319

Lohoff, F. W., Etemad, B., Mandos, L. A., Gallop, R., & Rickels, K. (2010). Ziprasidone Treatment of Refractory Generalized Anxiety Disorder. *Journal of Clinical Psychopharmacology*, *30*(2), 185–189. https://doi.org/10.1097/JCP.0b013e3181d21951

Lorenz, R. A., Jackson, C. W., & Saitz, M. (2010). Adjunctive use of atypical antipsychotics for treatment-resistant generalized anxiety disorder. *Pharmacotherapy*, *30*(9), 942–951. https://doi.org/10.1592/phco.30.9.942

Menza, M. A., Dobkin, R. D., & Marin, H. (2007). An open-label trial of aripiprazole augmentation for treatment-resistant generalized anxiety disorder [3]. *J Clin Psychopharmacol*, *22*(2), 207–210. https://doi.org/10.1097/01.jcp.0000248620.34541.bc

Milrod, B., Altemus, M., Gross, C., Busch, F., Silver, G., Christos, P., … Schneier, F. (2016). Adult separation anxiety in treatment nonresponders with anxiety disorders: Delineation of the syndrome and exploration of attachment-based psychotherapy and biomarkers. *Comprehensive Psychiatry*, *66*, 139–145. https://doi.org/10.1016/j.comppsych.2016.01.004

National Institute for Health and Clinical Excellence. (2011). *Generalised anxiety disorder and panic disorder in adults: management*. *CG113*. Manchester, United Kingdom.

Ociskova, M., Prasko, J., Latalova, K., Kamaradova, D., & Grambal, A. (2016). Psychological factors and treatment effectiveness in resistant anxiety disorders in highly comorbid inpatients. *Neuropsychiatric Disease and Treatment*, *12*, 1539–1551. https://doi.org/10.2147/NDT.S104301

Otto, M. W., Pollack, M. H., Penava, S. J., & Zucker, B. G. (1999). Group cognitive-behavior therapy for patients failing to respond to pharmacology for panic disorder: A clinical case series. *Behaviour Research and Therapy*, *37*(8), 763–770. https://doi.org/10.1016/S0005-7967(98)00176-4

Pallanti, S., & Quercioli, L. (2006). Resistant social anxiety disorder response to Escitalopram. *Clinical Practice and Epidemiology in Mental Health*, *2*(35). https://doi.org/10.1186/1745-0179-2-35

Patterson, B., & Van Ameringen, M. (2016). AUGMENTATION STRATEGIES FOR TREATMENT-RESISTANT ANXIETY DISORDERS: A SYSTEMATIC REVIEW AND META-ANALYSIS. *Depression and Anxiety*, *33*(8), 728–736. https://doi.org/10.1002/da.22525

Pollack, M. H. (2009). Refractory Generalized Anxiety Disorder. *The Journal of Clinical Psychiatry*, *70*(suppl 2), 32–38. https://doi.org/10.4088/JCP.s.7002.06

Pollack, M. H., Otto, M. W., Kaspi, S. P., Hammerness, P. G., & Rosenbaum, J. F. (1994). Cognitive behavior therapy for treatment-refractory panic disorder. *Journal of Clinical Psychiatry*, *55*(5), 200–205. Retrieved from http://www.embase.com/search/results?subaction=viewrecord&from=export&id=L24184829

Pollack, M. H., Simon, N. M., Zalta, A. K., Worthington, J. J., Hoge, E. A., Mick, E., … Oppenheimer, J. (2006). Olanzapine augmentation of fluoxetine for refractory generalized anxiety disorder: A placebo controlled study. *Biological Psychiatry*, *59*(3), 211–215. https://doi.org/10.1016/j.biopsych.2005.07.005

Rickels, K., Shiovitz, T. M., Ramey, T. S., Weaver, J. J., Knapp, L. E., & Miceli, J. J. (2012). Adjunctive therapy with pregabalin in generalized anxiety disorder patients with partial response to SSRI or SNRI treatment. *International Clinical Psychopharmacology*, *27*(3), 142–150. https://doi.org/10.1097/YIC.0b013e328350b133

Samuel, M., Zimovetz, E. A., Gabriel, Z., & Beard, S. M. (2011). Efficacy and safety of treatments for refractory generalized anxiety disorder: A systematic review. *International Clinical Psychopharmacology*, *26*(2), 63–68. https://doi.org/10.1097/YIC.0b013e328341bb4a

Simon, N. M., Hoge, E. A., Fischmann, D., Worthington III, J. J., Christian, K. M., Kinrys, G., & Pollack, M. H. (2006). An open-label trial of risperidone augmentation for refractory anxiety disorders. *Journal of Clinical Psychiatry*, *67*(3), 381–385. Retrieved from http://www.embase.com/search/results?subaction=viewrecord&from=export&id=L43556561

Simon, N. M., Otto, M. W., Worthington, J. J., Hoge, E. A., Thompson, E. H., LeBeau, R. T., … Pollack, M. H. (2009). Next-Step Strategies for Panic Disorder Refractory to Initial Pharmacotherapy. *The Journal of Clinical Psychiatry*, *70*(11), 1563–1570. https://doi.org/10.4088/JCP.08m04485blu

Snyderman, S. H., Rynn, M. A., Rickels, K., Snydermae, S. H., Rynn, M. A., Rickels, K., … Rickels, K. (2005). Open-label Pilot Study of Ziprasidone for Refractory Generalized Anxiety Disorder. *Journal of Clinical Psychopharmacology*, *25*(5), 497–499. https://doi.org/10.1097/01.jcp.0000177853.15910.de

Solbakken, O. A., & Abbass, A. (2015). Intensive short-term dynamic residential treatment program for patients with treatment-resistant disorders. *Journal of Affective Disorders*, *181*, 67–77. https://doi.org/10.1016/j.jad.2015.04.003

Solbakken, O. A., & Abbass, A. (2016). Symptom- and personality disorder changes in intensive short-term dynamic residential treatment for treatment-resistant anxiety and depressive disorders. *Acta Neuropsychiatrica*, *28*(05), 257–271. https://doi.org/10.1017/neu.2016.5

Starcevic, V. (2008). Treatment of panic disorder: Recent developments and current status. *Expert Review of Neurotherapeutics*, *8*(8), 1219–1232. https://doi.org/10.1586/14737175.8.8.1219

Stein, D. J. (2003). Algorithm for the pharmacotherapy of anxiety disorders. *Current Psychiatry Reports*, *5*(4), 282–288. Retrieved from http://www.embase.com/search/results?subaction=viewrecord&from=export&id=L38899220

Stein, D. J. (2004). Algorithms for primary care: An evidence-based approach to the pharmacotherapy of depression and anxiety disorders. *Primary Psychiatry*, *11*(6), 55–78. Retrieved from http://www.embase.com/search/results?subaction=viewrecord&from=export&id=L38879037

Stein, D. J., Baldwin, D. S., Bandelow, B., Blanco, C., Fontenelle, L. F., Lee, S., … Van Ameringen, M. (2010). A 2010 evidence-based algorithm for the pharmacotherapy of social anxiety disorder. *Current Psychiatry Reports*, *12*(5), 471–477. https://doi.org/10.1007/s11920-010-0140-8

Stein, D. J., Kasper, S., Matsunaga, H., Osser, D. N., Stein, M. B., Van Ameringen, M., … Zhang, M. (2001). Pharmacotherapy of social anxiety disorder: an algorithm for primary care - 2001. *Primary Care Psychiatry*, *7*(3), 107–110. https://doi.org/10.1185/135525701317094368

Tesar, G. E., & Rosenbaum, J. F. (1986). Successful use of clonazepam in patients with treatment-resistant panic disorder. *Journal of Nervous and Mental Disease*, *174*(8), 477–482. Retrieved from http://www.embase.com/search/results?subaction=viewrecord&from=export&id=L16040140

Van Ameringen, M., Mancini, C., & Patterson, B. (2009). Pharmacotherapy for social anxiety disorder and specific phobia. In M. M. Antony, M. B. Stein, M. M. Antony (Ed), & M. B. Stein (Ed) (Eds.), *Oxford handbook of anxiety and related disorders.* (pp. 321–333). New York, NY, US: Oxford University Press. Retrieved from http://search.ebscohost.com/login.aspx?direct=true&db=psyh&AN=2008-13193-024&site=ehost-live

Worthington III, J. J., Kinrys, G., Wygant, L. E., & Pollack, M. H. (2005). Aripiprazole as an augmentor of selective serotonin reuptake inhibitors in depression and anxiety disorder patients. *International Clinical Psychopharmacology*, *20*(1), 9–11. https://doi.org/10.1097/00004850-200501000-00002

Yoshinaga, N., Matsuki, S., Niitsu, T., Sato, Y., Tanaka, M., Ibuki, H., … Shimizu, E. (2016). Cognitive behavioral therapy for patients with social anxiety disorder who remain symptomatic following antidepressant treatment: A randomized, assessor-blinded, controlled trial. *Psychotherapy and Psychosomatics*, *85*(4), 208–217. https://doi.org/10.1159/000444221

Zoun, M. H. H., Koekkoek, B., Sinnema, H., Muntingh, A. D. T., van Balkom, A. J. L. M. A. J. L. M., Schene, A. H., … Spijker, J. (2016). Effectiveness and cost-effectiveness of a self-management training for patients with chronic and treatment resistant anxiety or depressive disorders: design of a multicenter randomized controlled trial. *BMC Psychiatry*, *16*(1). https://doi.org/10.1186/s12888-016-0927-1
